# Targeted Inhibition of Photosystem II Electron Transport Using Bioherbicide-Loaded Ultrasmall Nanodevices

**DOI:** 10.1021/acsomega.5c07085

**Published:** 2025-11-17

**Authors:** Montcharles S. Pontes, Leandro O. Araujo, Jaqueline S. Santos, José Luiz da Silva, Thaiz B.A.R. Miguel, Emilio C. Miguel, Sandro M. Lima, Luis H. C. Andrade, Gilberto J. Arruda, Jean-Claude M’Peko, Samuel L. Oliveira, Renato Grillo, Anderson R. L. Caires, Etenaldo F. Santiago

**Affiliations:** † Plant Resources Study Group, Center for Natural Resources Study (CERNA), 67708 Mato Grosso do Sul State University (UEMS), Dourados, Mato Grosso do Sul 79804-970, Brazil; ‡ Optics and Photonics Group, SISFOTON Lab, Institute of Physics, Federal University of Mato Grosso do Sul (UFMS), Campo Grande, Mato Grosso do Sul 79070-900, Brazil; § Genetics Department, Luiz de Queiroz College of Agriculture (ESALQ), University of São Paulo (USP), Piracicaba, São Paulo 13418-900, Brazil; ∥ Department of Analytical, Physico-Chemical and Inorganic Chemistry, Institute of Chemistry, São Paulo State University (UNESP), Araraquara 14800-060, Brazil; ⊥ Laboratory of Biomaterials, Department of Metallurgical and Materials Engineering, 28121 Federal University of Ceará (UFC), Fortaleza, Ceará 60020-181, Brazil; # São Carlos Institute of Physics, University of São Paulo (USP), São Carlos, São Paulo 13566-590, Brazil; ¶ Environmental Nanochemistry Group, Department of Physics and Chemistry, São Paulo State University (UNESP), Ilha Solteira, São Paulo 15385-000, Brazil

## Abstract

Usnic acid (UA) is a promising bioherbicide with a mode of action targeting photosystem II (PSII) inhibition. This study investigates the enhancement of UA’s herbicidal efficacy through a novel nanoformulation using ultrasmall superparamagnetic iron oxide nanoparticles (USPIONs) as a smart delivery system. USPIONs presenting a sub-10 nm mean particle diameter were synthesized and thoroughly characterized for agricultural applications, with the objective of improving UA delivery and achieving controlled release. The basal release kinetic results revealed that c.a. 1086 min were required to release 50% of the UA release (t_50%_) and when nanoparticle solution was exposed to an external alternating magnetic field (AMF) exposure, the time to 50% UA release (t50%) was about 41.03 min. *In vivo* chlorophyll fluorescence analysis revealed that the nanoenabled formulation enhanced PSII inhibition, enhancing suppression of electron flow at the quinone A (Q_A_) to quinone B (Q_B_) interface. The uncapped and oleic acid-capped USPIONs exhibited reduced Fv/Fm values, to 18.93% and 27.34%, respectively, compared to free usnic acid. Furthermore, gene expression analysis showed a 2.5-fold upregulation in the photosynthetic genes *psbA* and *petA*, compared to that in untreated control plants, indicating a robust physiological response. Enzyme assays demonstrated an upregulation in activities of superoxide dismutase and catalase (SOD, CAT) in treated lettuce leaves, underscoring the induction of oxidative stress. Molecular docking simulations highlighted the preferential binding of UA within the Q_B_-binding domain, suggesting a strong interaction potential at the catalytic site. Additionally, USPIONs were predicted to interact near the center of the D1 protein. These findings indicate that USPIONs enhance the PSII-inhibitory action of UA relative to its nonloaded form, supporting their feasibility as targeted bioherbicide carriers pending broader agronomic and environmental validation.

## Graphical abstract

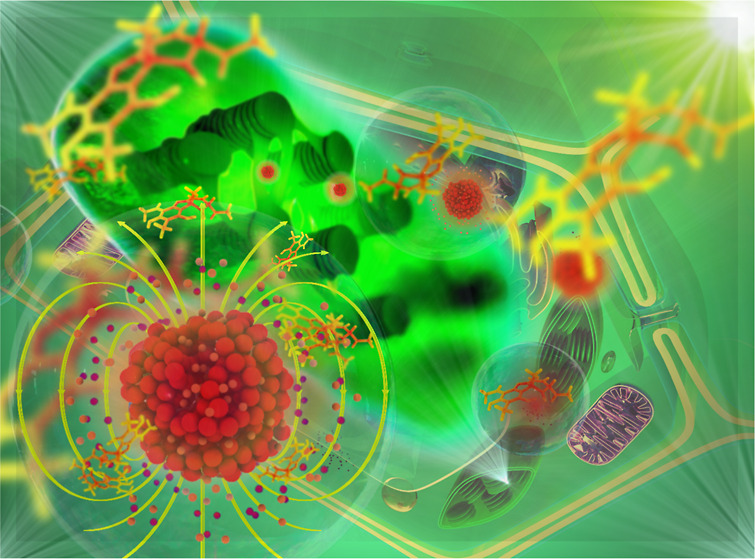

## 1 Introduction

Nanoengineered carriers can modulate bioactive distribution, cellular uptake, and targeted accumulation, potentially minimizing off-target exposure and environmental burdens associated with conventional agrochemicals.^1–3^ Ultrasmall superparamagnetic iron oxide nanoparticles (USPIONs) are widely utilized in multiple roles, including MRI contrast enhancement for clinical diagnostics, vehicles for molecular payloads, and platforms for nanosensing.^4–10^ USPION size and surface attributes can be engineered to match specific application requirements,^11^ providing significant advantages across fields. These particles generate localized heating under alternating magnetic fields and can be magnetically recovered from intricate matrices.^10,12^

Utilizing USPIONs as localized heating sources represents a leading strategy for targeted and smart chemical cargo release.^13^ Magnetic hyperthermia treatments, induced by alternating magnetic fields, aim to increase the temperature of USPIONs locally, thereby triggering the release of active cargo within *in vivo* environments after successfully overcoming biological barriers^14,15^ Despite advancements in smart, controlled, and targeted on-demand pesticide release technologies,^16^ the use of USPIONs as delivery vehicles for pesticides and biopesticides in nanoenabled agriculture remains limited. However, this controlled-release mechanism can be realistically implemented in localized treatment zones using portable electromagnetic devices operating in a manner analogous to precision spraying systems already established in modern agriculture. This approach offers the advantage of spatially and temporally controlled bioherbicide release, potentially reducing the overall chemical inputs while enhancing targeted efficacy in specific crop areas.

Although emerging natural molecules have been reported as potential herbicidal agents acting by impairing electron transport at photosystem II (PSII).^17–20^ Biogenic molecules frequently exhibit accelerated soil degradation kinetics relative to synthetic analogs, reducing persistence-related environmental risks.^20–22^ The lichen-derived secondary metabolite, usnic acid (UA) [2,6-diacetyl-7,9-dihydroxy-8,9*b*-dimethyl-1,3­(2*H*,9*bH*)-dibenzo-furandione], possesses recognized phytotoxic properties and herbicidal activity against C_3_ plants, C_4_ plants, and algae,^23–25^ particularly due to its capacity to hinder electron transport at the PSII site.^18^ Few studies have documented the loading of UA into nanostructured materials, including polymeric and metal oxide nanoparticles, mainly for biomedical applications.^11^ Currently, the precise mechanism by which nanoenabled UA’s affect photosynthesis remains poorly characterized, despite the growing focus on plant-nanobiopesticide interactions.

Previous studies demonstrate that UA exerts its phytotoxic effects by targeting the Q_B_ pocket within the D1 subunit, thereby disrupting electron flow.^18^ Consequently, the electron transfer between Q_A_ and Q_B_ is blocked, recapitulating the mode of action of several commercial PSII herbicides. Recently, our group explored ultrasmall SPIONs as delivery vehicle for the bioherbicide usnic acid, aiming to enable more sustainable and precise agricultural practices with reduced environmental impact, particularly regarding soil microbial health during weed management processes.^13^ However, the mechanisms underlying their enhanced action remain unclear. Building upon this critical knowledge gap, we herein investigated the PSII inhibition mechanisms of nanoenabled usnic acid in *Lactuca sativa* (L). (a representative C3 model). Our results are designed to clarify the comparative phytotoxic effects of the nanoformulation versus its conventional counterpart, thereby providing essential data for the rational design of safe and sustainable nanobiopesticides for precision agriculture.

## 2 Experimental Procedure

### 2.1 Chemicals

Reagents included FeCl_3_·6H_2_O, FeCl_2_·4H_2_O, acetic acid, usnic acid, and oleic acid (Sigma-Aldrich, USA). TRIzol and RT-qPCR kits were purchased from Thermo Fisher Scientific (USA). All chemical materials were of analytical grade and used as received; distilled water served as the solvent for all of the prepared solutions.

### 2.2 Synthesis and Characterization of Cargo-Loaded USPIONs

The ultrasmall SPIONs were synthesized via an alkaline coprecipitation route (modified protocol).^10^ A mixture of Fe­(III)/Fe­(II) salts (1.0/1.6 g) was dissolved in 200 mL water, precipitated with 8 mL 25% NaOH solution carried out with the presence or absence of 500 mg of UA for surface functionalization. The mixture was held at 50 °C with stirring for 10 min before the capping agent (oleic acid). The final reaction proceeded at 80 °C for 1 h. The resulting magnetic nanoparticles were rigorously purified through sequential washing cycles with water and ethanol. The USPIONs (uncapped ultrasmall superparamagnetic nanoparticles), USPIONs/UA (usnic acid adsorbed as cargo onto ultrasmall superparamagnetic nanoparticles), USPIONs@OA (oleic acid-capped ultrasmall superparamagnetic nanoparticles), and USPIONs@OA/UA (usnic acid adsorbed as cargo onto ultrasmall superparamagnetic nanoparticles capped with oleic acid) were then dried.

Particle morphology and size distribution were assessed by FEG-SEM (JSM-7500F, JEOL) and by TEM at 200 kV (CM200, Philips) for USPIONs, USPIONs/UA, USPIONs@OA, and USPIONs@OA/UA. Crystallographic profiles were obtained by X-ray diffraction using a Rigaku MiniFlex 600 diffractometer (Rigaku, Tokyo) operating in Bragg–Brentano geometry with Cu Kα radiation (λ = 1.5406 Å) and a HyPix-400 MF 2D HPAD detector. The average crystallite size for each sample was estimated using Debye–Scherrer’s approach [eq 01]­

01 $$D=\frac{K\lambda }{\beta \;\cos\;\theta }$$

where *K* (Scherrer factor), λ (X-ray wavelength, 1.5406 Å), β (fwhm in radians), and θ (Bragg angle) are the parameters used in the calculation.

Crystallinity was primarily confirmed by XRD, revealing reflections typical of inverse-spinel magnetite (Fe_3_O_4_) (see Table S1 and Figure S4, Supporting Information); all crystallinity assessments are based on XRD and the agreement of average crystallite size with TEM measurements.

The zeta potential was determined for USPIONs, USPIONs/UA, USPIONs@OA, and USPIONs@OA/UA using the Dynamic Light Scattering (DLS) model Zetasizer Nano ZS (Malvern). For sample preparation, 0.2 g of each nanoparticle formulation was dispersed in 1 nM mL of NaCl electrolyte in 200 mL of distilled water. Dispersions were probe-sonicated at 80 W for 10 min to promote deagglomeration. After an initial 24 h settling period, the supernatant was carefully collected and subjected to zeta potential analysis.

UA release was studied in a two-chamber dialysis arrangement; donor and acceptor cells were divided by a 10 kDa molecular-weight-cutoff membrane. Release kinetics were assessed under two distinct conditions: (i) passive release at room temperature under magnetic stirring and (ii) stimuli-responsive release via magnetic heat induction. The alternating magnetic field was applied with the following settings: 36.28 V, 5.68 A, 207 W, and duty cycle 63% (3.04 Oe·h). Experiments were performed at an ambient temperature. UA release was quantified according to [eq 02]­

02 $$cargo\;release\;\left(\%\right)=\frac{M_{i}}{M_{t}}\times 100\%$$

where, *M*_i_ denotes the concentration of released chemical cargo at i time and *M*_t_ is the total initial chemical cargo content added to the USPION nanocarrier solution.

### 2.3 Plant Materials and Nanobioherbicide Exposure

Seeds of *L. sativa* L. (Crespa cultivar; Isla Sementes, Porto Alegre, Brazil) were obtained from a commercial supplier. Lettuce seeds were germinated and grown on polystyrene boxes for 30 days. After the growth period, intact leaves were selected and rinsed with sterile distilled H_2_O, and the surface was exposed by spraying to USPIONs, USPIONs/UA, USPIONs@OA, and USPIONs@OA/UA solutions at 500 ppm to investigate their bioherbicidal effect. Sterile distilled H_2_O was used as the negative control, and UA at same amount was used as the positive control. The effects were evaluated after 7 days of exposure. The concentration used in this study was selected based on a previous dose-dependent assay, where the inhibition of photosystem II by UA was evaluated through changes in the dark-adapted photochemical quantum yield of photosystem II (Fv/Fm) values (Figure S1, Supporting Information).

### 2.4 Insights into the Mode of Action through Photosystem II Inhibition Assays

#### 2.4.1 Chlorophyll Content

Chlorophylls were quantified from 50 mg of fresh lettuce tissues. Samples were macerated, extracted in 10 mL of ice cold 99% ethanol, and then centrifuged at 12.000*g* for 20 min. Absorbance at 649 and 665 nm was recorded and concentrations of Chl *a*, Chl *b*, and total chlorophyll were calculated according to Lichtenthaler and Wellburn.^26^ Measurements were taken at room temperature on a UV-5200 spectrophotometer (Global Trade Technology). Results are reported as mg g^–1^ fresh weight and calculated using the following equations

03 $$Chl\;a=\left(13.95\times {Abs}_{665nm}\right)-\left(6.88\times {Abs}_{649nm}\right)$$

04 $$Chl\;b=\left(24.96\times {Abs}_{649nm}\right)-\left(7.32\times {Abs}_{665nm}\right)$$

05 $${Chl}_{Total}=\left(Chl\;a\;content\right)+\left(Chl\;b\;content\right)$$

#### 2.4.2 Chlorophyll *a* Fluorescence Induction

To probe PSII electron transport and energy dissipation in lettuce, *in vivo* chlorophyll *a* fluorescence (OJIP) was recorded 168 h post-treatment on dark-adapted leaves using a hand-held PEA fluorometer (Hansatech Instruments, UK). It should be noted that these measurements represent *in vivo* chlorophyll fluorescence from intact photosynthetic tissues. The measurement was performed by using red light excitation (Ex λ = 630 nm) at an intensity of 3500 μmol m^–2^ s^–1^. The data obtained were used to calculate photochemical and biophysical parameters according to the JIP-test equations.^27^ Chlorophyll fluorescence intensities were assessed as follows: (i) minimal fluorescence intensity at 20 μs, corresponding to all PSII reaction centers (RCs) being open (O step or F_0_); (ii) intermediate fluorescence intensities at 150 μs (L step), 300 μs (K step or K-band), 2 ms (J step), and 30 ms (I step); and (iii) maximal fluorescence intensity when all PSII RCs are closed (P step).^27^ A total of 25 measurements were recorded for each treatment, and all analyses were conducted at room temperature.

##### 2.4.2.1 Specific Energy Fluxes per Q_A_-Reduced PSII Reaction Center

The specific energy fluxes per reaction center (RC) were analyzed to assess the influence of the magnetic nanobiopesticide on the photosynthetic machinery, electron transport processes, and light energy conversion efficiency. These parameters included the light absorption flux per active RC (ABS/RC), representing the ratio between active and inactive RCs; the trapping of excitation energy resulting in a photochemical reaction in Q_A_ (TR_0_/RC); the dissipation of excess energy not captured by an RC, occurring through heat release, fluorescence emission, or energy transfer to other pathways (DI_0_/RC); the electron transport flux (ET_0_/RC); and the electron flux associated with the reduction of the PSI end acceptor (RE_0_/RC).^28–30^ The specific energy fluxes per RC were calculated as follows

06 $$\frac{ABS}{RC}=M_{0}\left(\frac{1}{V_{J}}\right)\left(\frac{1}{F_{v}{/F}_{m}}\right)$$

07 $$\frac{{TR}_{0}}{RC}=M_{0}\left(\frac{1}{V_{J}}\right)$$

08 $$\frac{{DI}_{0}}{RC}=\frac{ABS}{RC}-\frac{{TR}_{0}}{RC}$$

09 $$\frac{{ET}_{0}}{RC}=M_{0}\left(\frac{1}{V_{J}}\right)\left(1-V_{J}\right)$$

10 $$\frac{{RE}_{0}}{RC}=\left(\frac{1-V_{I}}{1-V_{J}}\right)M_{0}\left(\frac{1}{V_{J}}\right)$$

##### 2.4.2.2 Fraction of Q_A_-Reducing RCs per PSII Antenna Chlorophyll

The density of active reaction centers (RCs) on a chlorophyll basis was determined as the proportion of Q_A_-reducing RCs (active) relative to that of the PSII antenna chlorophyll (Chl). This parameter was calculated using the following equation [eq 03]­

11 $$\frac{RC}{ABS}=\frac{{Chl}_{RC}}{{Chl}_{Antenna}}=\frac{\gamma_{RC}}{\left({1-\gamma }_{RC}\right)}=\left[\frac{F_{J}-F_{O}}{4\left(F_{300\mu s}-F_{0}\right)\left(F_{V}-F_{O}\right)}\right]$$

where, the factor 4 represents the conversion used to express the initial fluorescence rise per millisecond.

#### 2.4.3 Real-Time Quantitative PCR (RT-qPCR) Analysis of Photosynthetic Genes

Total RNA was extracted from lettuce leaves with TRIzol, following the manufacturer’s protocol. The RNA concentration and purity were assessed on a NanoDrop μ-volume UV–vis spectrophotometer (Thermo Fisher Scientific). Residual genomic DNA was removed by dsDNase treatment (5 μg of RNA with 4 U at 37 °C for 2 min). One microgram of DNase-treated RNA was reverse-transcribed using the Maxima First Strand cDNA Synthesis Kit for RT-qPCR (Thermo Fisher Scientific). Reactions (reaction mix, Maxima Enzyme Mix, nuclease-free water) were incubated 10 min at 25 °C and 15 min at 50 °C and then heated to 85 °C for 5 min to terminate; cDNA was stored at −20 °C until use. Photosynthesis-related genes were identified via the Lettuce Genome Resource (LGR) database (https://lgr.genomecenter.ucdavis.edu). Primers were designed in Primer3Plus (https://primer3plus.com). Additional primers were developed for genes associated with photosynthesis-related pathways (Table 1), following Mariz-Ponte et al.^31^ The selected genes are primarily involved in the mechanisms of photosynthetic light capture and energy conversion.

**Table 1:** Primer Sequences Used for Amplification of Photosynthesis-Related Genes in *L. sativa* Studied

gene name—definition	accession number	5′-forward/3′-reverse
*psbA*—photosystemII D1 protein	NC_007578.1:c1540–479	F: GTGTAGCTTGTTACATGGGTCGT
		R: TCCTAGAGGCATACCATCAGAAAAG
*petA*—cytochrome*f*	NC_007578.1:62045–63007	F: GATACGAAATAACCATAGCGGATG
		R: ATCCCTGGCTTCGGAAAG
*petB*—cytochrome *b*6	NC_007578.1:74837–76254	F: ACAGGTGTGGTTCTGGGTGT
		R: GTGGATTGTCCCACACTAGCA
*psaA*—photosystemI P700 chlorophyll *a* apoprotein A1	NC_007578.1:c41453–39201	F: ATGGCTAAGCGATCCGACT
		R: TCCAGATGCTCGCCAAAT

RT-qPCR reactions (10 μL) contained 2.5 μL of cDNA, 5 μL of iTaq Universal SYBR Green Supermix (Bio-Rad), and primers (2.5 μL, 10 μM). Amplifications were run on a CFX96 Real-Time PCR system (Bio-Rad) with the following program: 95 °C for 1 min; 40 cycles of 95 °C for 3 s, and 60 °C for 30 s. Melting curves were acquired from 65 to 95 °C with 0.5 °C steps every 10 s. Relative transcript abundance was computed by the 2^–^ΔΔCt method, as described by Livak and Schmittgen.^32^

### 2.5 Molecular Docking Simulation

#### 2.5.1 Protein Homology

The FASTA sequence of the photosystem II D1 (psbA) protein from *L. sativa* was obtained from UniProt (UniProt: P69557). The amino acid sequence was used to predict the structure using SwissModel server (https://swissmodel.expasy.org). The pdb file of generated model of target protein is shown in Figure S2 Supporting Information.

#### 2.5.2 Data Set of Ligands and Structure Preparation

The usnic acid structure was created in ChemDraw 14.0 and subsequently optimized through MM2 energy minimization. The molecular structure was saved as an MDL mol file (Figure S3A Supporting Information). The crystallographic data extracted from Fe_3_O_4_ nanoparticles were used to generate the 3D structure of ultrasmall superparamagnetic iron oxide nanoparticles (USPIONs). The Nanocrystal software tool was used for the construction of nanoparticle structure.^33^ A spherical nanoparticle model was generated by uploading the Fe_3_O_4_ crystal structure in CIF format using Miller indices and corresponding minimum surface energies derived from experimental data. A nanoparticle was generated with a particle size of 1.0 nm and 408 atoms (Figure S3B Supporting Information) according to the Wulff morphology construction method.

#### 2.5.3 USPION Binding Predictions

The binding prediction of USPIONs with PSII D1 protein of *L. sativa* was built to predict NP-binding residues, and the molecular docking studies were performed using the platform Patchdock. All visualizations and conformations resulting from USPIONs and PSII D1 interactions were examined using the Chimera X software package, version 1.2.^34^ The ten best scores of amino acid residues and USPION interactions were selected for evaluation in this study.

#### 2.5.4 Usnic Acid Docking onto PSII D1: Searching Binding Residues at the Q_B_ Site

Ligand conformers were energy-minimized prior to docking using the program’s internal force field. Following geometry optimization, the UA was docked within a predefined region using the MMFF94 force field. A grid box of 40 × 40 × 40 points along the *x*, *y*, and *z* axes with a spacing of 0.486 Å was centered at coordinates *X* = – 39.00, *Y* = 5.00, and *Z* = – 20.00.

### 2.6 Stress-Related Antioxidant Enzyme Activity

Superoxide dismutase (SOD) was assayed via inhibition of NBT photoreduction.^35^ Reactions (5 mL) contained 50 mM Na_2_CO_3_ (pH 10.0), 13 mM methionine, 0.025% (v/v) Triton X-100, 63 μM NBT, 1.3 μM riboflavin, and an aliquot of enzyme extract; mixtures were illuminated for 15 min at PPFD ≈ 380 μmol m^–2^ s^–1^, with a nonilluminated blank. One unit of SOD activity corresponded to the enzyme amount, producing 50% inhibition of NBT reduction, monitored at 560 nm. Catalase (CAT) activity was determined by the decrease in H_2_O_2_ absorbance at 240 nm over 5 min in 25 mM Tris­(acetate) buffer (pH 7.0) containing 0.8 mM EDTA-Na and 20 mM H_2_O_2_. Total volume was 3 mL; assays were carried out in triplicate at room temperature (≈25 °C). Catalase (CAT) activity was determined by the decrease in H_2_O_2_ absorbance at 240 nm over 5 min in 25 mM Tris–acetate buffer (pH 7.0) containing 0.8 mM EDTA-Na and 20 mM H_2_O_2_.^22^ Total volume was 3 mL; assays were carried out in triplicate at room temperature (≈25 °C).

Glutathione peroxidase activity was measured using a modified protocol developed by Flohe et al.,^36^ in which H_2_O_2_ served as the substrate. Crude enzyme extracts were prepared in prechilled KNaHPO_4_ buffer (pH 7.0) using a homogenizer; the supernatant obtained after centrifugation (1100*g*, 10 min) was used for assays. For each reaction, 0.2 mL of extract was mixed with 0.4 mL of reduced GSH (0.1 mM) and 0.2 mL of KNaHPO_4_ (0.067 M). A blank without enzyme was prepared in parallel. After preincubation at 25 °C for 5 min, the reaction was started by adding 0.2 mL H_2_O_2_ (1.3 mM) and allowed to proceed for 10 min. Reactions were stopped with 1 mL 1% trichloroacetic acid and kept on ice for 30 min, then centrifuged (1100*g*, 10 min). An aliquot of the supernatant (0.48 mL) was transferred to a cuvette; for color development, 2.2 mL 0.32 M Na_2_HPO_4_ and 0.32 mL 1.0 mM DNTB were added, and absorbance was read at 412 nm after 5 min. Activity was expressed as the decrease in GSH relative to the nonenzymatic control.

### 2.7 Data Analysis

A CRD (completely randomized design) was adopted, assigning treatments at random and using three replicates per treatment. A two-factor ANOVA was applied, followed by Fisher’s LSD (protected by a significant omnibus F) for mean separation at *p* < 0.05. Optical spectroscopy data were compared using two-tailed Student’s *t* tests with a significance threshold of *p* < 0.05. Results are displayed as mean values with standard error bars, and analyses were conducted in RStudio.

Global connectance (Cg) was used to quantify modulation in the photosynthetic network from selected photochemical signals, representing the mean strength of pairwise connections after Fisher *z*-transformation of the correlation coefficients. PSII energy fluxes in the energy cascade for the events was defined following Strasser’s framework and used to compose the photochemical network.^27^ Network connectance analysis was computed as the means of absolute *z*-values across the specified variable pairs, following the Amzallag connectance approach to assess systemic coordination.^33,37^ Global connectance (Cg) was derived from a correlation network built with Pearson coefficients (*r*) among energy fluxes parameters, normalizing each coefficient via Fisher’s *z*-transformation: *z* = ln­(1 + |*r*|)/(1 – |*r*|).^30^ Cg was obtained by averaging |*z*| across all eligible pairs after Fisher’s transformation, yielding the network’s average connection intensity. This procedure follows the Amzallag framework for network connectance.^37^

## 3 Results and Discussion

### 3.1 Synthesis and Characterization of Magnetic Nanostructures for Cargo Delivery

A one-step alkaline coprecipitation route was adopted to obtain magnetite at high yield using a protocol amenable to scale-up.^10^ Oleic acid was applied as a surface capping agent to stabilize the nanoparticles in organic media and to promote a strong affinity with the selected cargo. UA was loaded prior to OA treatment, and the product was recovered by magnetic separation. In this study, we compared the physiological responses to USPIONs (uncapped), USPIONs@OA (OA-capped), USPIONs/UA (UA-adsorbed), and USPIONs@OA/UA (OA-capped with UA) (Figure 1I-L). Transmission electron microscopy images showed that USPION, USPIONs@OA, USPIONs/UA, and USPIONs/UA@OA nanomaterials (Figure 1A–D) were magnetite nanoparticles with crystalline structures and average particle sizes of 5.63 ± 1.6, 7.72 ± 2.3, 5.22 ± 4.4, and 7.21 ± 3.5 nm, respectively (Figure 1I–L). Kernel density estimation plots reveal distinct distribution patterns across the nanoparticle formulations, with USPIONs and USPIONs/UA@OA exhibiting relatively homogeneous particle size distributions centered around their respective mean values, while USPIONs@OA and USPIONs/UA demonstrate broader, more heterogeneous distributions indicative of increased dispersity following surface functionalization and bioherbicide loading processes. Elemental analysis by EDS revealed the expected signals for iron, oxygen, and chlorine in every nanoparticle formulation, validating the successful synthesis and functionalization of magnetite-based nanostructures (Figure 1E–H). The XRD pattern indicates a single-phase material, with reflections consistent with inverse-spinel magnetite (Fe_3_O_4_) (Figure S4 Supporting Information). The average crystallite sizes were 5.96 ± 0.7, 8.33 ± 1.2, 7.73 ± 0.5, and 7.51 ± 0.9 nm for USPIONs, USPIONs@OA, USPIONs/UA, and USPIONs@OA/UA, respectively. These values fall within the single-domain regime and agree with TEM-derived size distributions (Table S1 Supporting Information). The assignment of crystalline structure USPIONs was based on XRD analysis. Additionally, the dislocation density (δ) showed that USPIONs, USPIONs@OA, USPIONs/UA, and USPIONs/UA@OA nanostructures present δ values of 2.81 × 10^–4^, 1.44 × 10^–4^, 1.84 × 10^–4^, and 1.77 × 10^–4^ nm^2^, respectively (Table S1 Supporting Information). Dislocations are significant contributors to plastic deformations in metals. Our findings indicate that the incorporation of a capping agent and chemical cargo onto the nanoparticles induces subnanostructural changes.

**Figure 1:**
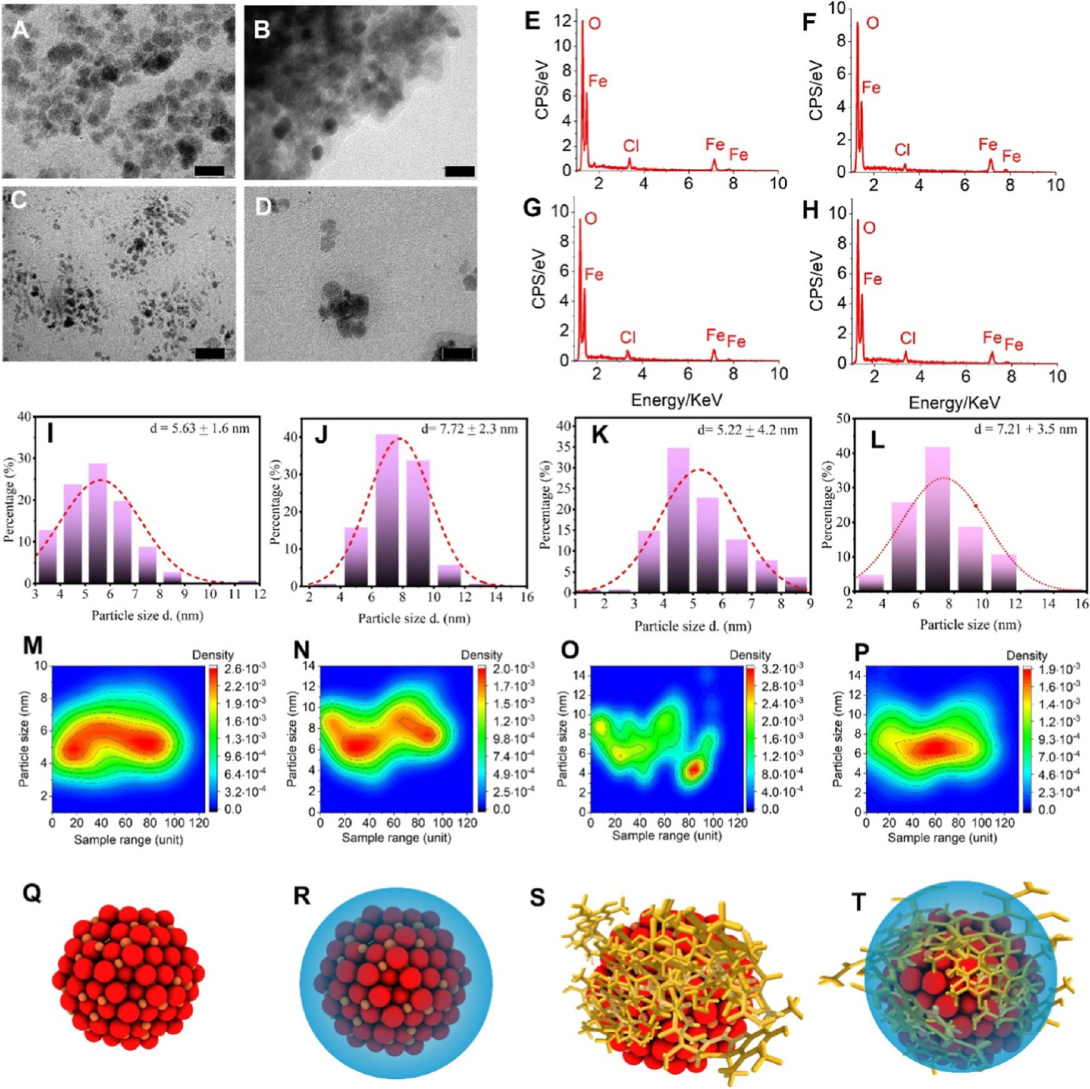
Characterization of usnic acid-loaded crystalline magnetite nanoparticles (USPIONs). (A–D) TEM images showing morphology and particle distribution of USPIONs (A), USPIONs@OA (B), USPIONs/UA (C), and USPIONs/UA@OA (D). (E–H) EDS spectra confirming elemental composition of USPIONs (E), USPIONs@OA (F), USPIONs/UA (G), and USPIONs/UA@OA (H). (I–L) Particle size distribution histograms obtained from statistical analysis of >100 particles using ImageJ software for USPIONs (I), USPIONs@OA (J), USPIONs/UA (K), and USPIONs/UA@OA (L). (M–P) Kernel density estimation plots demonstrating size distribution patterns and sample homogeneity/heterogeneity for USPIONs (M), USPIONs@OA (N), USPIONs/UA (O), and USPIONs/UA@OA (P). (Q–T) Schematic illustration of the nanostructures: USPIONs (Q), USPIONs@OA (R), USPIONs/UA (S), and USPIONs/UA@OA (T), respectively. Scale bar = 10 nm. Created by the authors.

As shown in Figure 2A,B, the release kinetics were assessed in two ways: (i) first, a basal release kinetic assay using a dialysis bag to establish an acceptor–donor relationship and (ii) release triggered by magnetic heat induction. The usnic acid release assays produced time-dependent profiles. This assay quantified the percentage release of the active ingredient, noting that a fraction of usnic acid remained in the donor compartment in both experiments (Figure 2C,D), accounting for approximately 40% in the basal release assay and 25% in the magnetic induction heating assay. The release profile revealed that c.a. 1086 min were required to release 50% of the UA release (t_50%_) (Figure 2C). The release rate of USPIONs/UA@OA was prompt increased when nanoparticle solution was exposed to an external alternating magnetic field (AMF), and the time required to release 50% of the UA (t_50%_) was c.a. 41.03 min (Figure 2D). When exposed to an AMF, magnetic nanoparticles may generate heat primarily via hysteresis losses in ferri/ferromagnetic particles and via Néel relaxation in superparamagnetic particles.^6^ Therefore, the irradiation of lipid-capped, bioherbicide-loaded nanostructures with an alternating magnetic field is expected to trigger a nanoparticle heating response. This response is expected to cause a transition in the lipid shell, thereby releasing the encapsulated chemical cargo from the nanoparticles. The release rate of USPIONs/UA@OA was prompt increased when nanomaterial solution was exposed to an external AMF, and the time required to release 50% of the UA (t_50%_) was c.a. 41.03 min (Figure 2D). In a 120 min control conducted without the alternating magnetic field, UA release from the magnetic nanoparticles was negligible (mean ± standard deviation = 0.8 ± 0.75%). The release mechanism likely arises from the combined effects of magnetite nanoparticle heating under an AMF and thermoresponsive transition of the oleic acid capping layer, which together promote payload desorption. This heating can increase the local temperature, promoting the release of the encapsulated content. Also, the OA capping agent is thermosensitive, meaning that its physical properties (such as viscosity or solubility) can change with temperature. The increase in temperature may induce a phase transition of the OA, resulting in the opening of release channels and allowing the active ingredient to be released.

**Figure 2:**
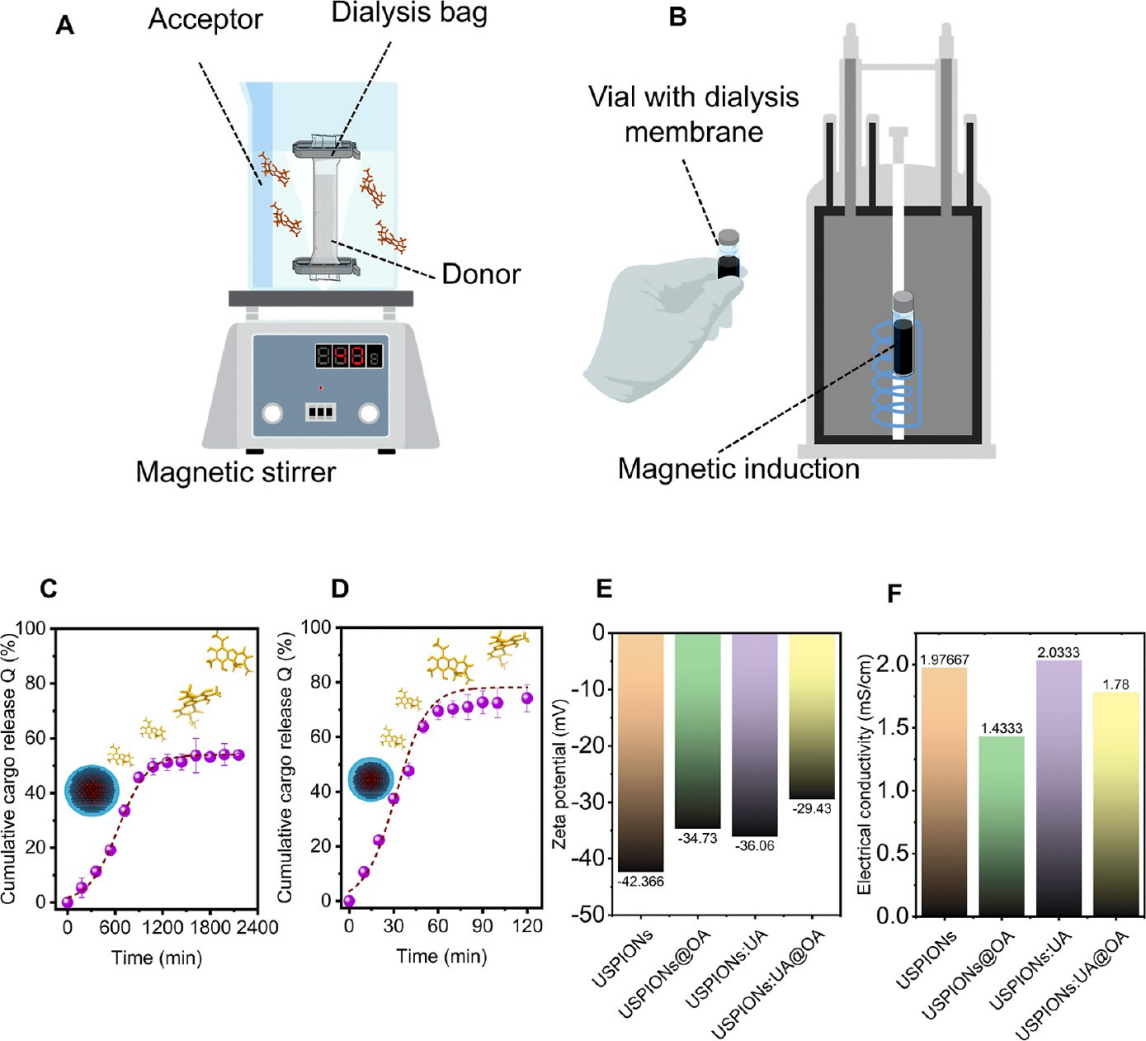
The schematic illustration of the release kinetics assay for (A) basal release study and (B) magnetic heat induction release study. Cumulative release rate profiles from (C) basal release study and (D) magnetic heat induction release study for USPIONs/UA@OA. (E) Zeta potential and (F) electrical conductivity of nanostructures used in this study, for USPIONs, USPIONs@OA, USPIONs/UA, and USPIONs/UA@OA, respectively. Figures 2A,B illustration created with BioRender.com. Created by the authors.

The surface charge evaluated by the zeta potential (ζ-potential) exhibited negative values (Figure 2E) for all particles used in this study. However, the ζ-potential of the coated particles is less negative compared to that of the bare particles, indicating that UA and OA can increase the surface charge of the particles. Despite this short increase, USPIONs@OA, USPIONs/UA, and USPIONs/UA@OA still presents a highly negative ζ-potential. Similar results are observed by Shete et al.^38^ with Fe_3_O_4_ nanoparticles capped with oleic acid target for biomedical applications. A decrease in negative surface charge may result from binding of UA or OA at the nanoparticle interface, which modifies exposed functional groups and ionic screening.^9,10^ Thus, this would likely increase the electrostatic repulsion and attraction between USPIONs, UA, and OA, potentially affecting the coalescence of the chemical cargo. Also, the conductivities of USPIONs, USPIONs@OA, USPIONs/UA, and USPIONs/UA@OA were determined. The values of conductivity of USPION, USPIONs@OA, USPIONs/UA, and USPIONs/UA@OA were observed to be c.a. 1.97 mS/cm, 1.43 mS/cm, 2.04 mS/cm, and 1.78 mS/cm, respectively (Figure 2F). This likely reflects the freer motion of Fe-derived ions (ionic release), UA, and OA anions/cations in the medium. The higher conductivity of USPIONs/UA and USPIONs/UA@OA compared with USPIONs@OA is probably due to long-range conductive pathways facilitated by UA molecules and/or coordination interactions between UA and Fe^2+^/Fe^3+^ that enhance charge transport.^39,40^

### 3.2 Mode of Action of Magnetic Bioherbicide Nanoparticle

#### 3.2.1 Chlorophyll Content

Many herbicides that inhibit photosynthesis may affect the synthesis of chlorophyll and hemes. The chlorophyll *a* (Chl *a*) content significantly decreased in lettuce leaves with UA treatment (Figure 3A). The Chl *a* level did not differ significantly across the nanoenabled bioherbicide formulations. On the other hand, chlorophyll *b* (Chl *b*) and total chlorophyll content (Chl) were reduced by USPIONs/UA@OA, compared with ionic usnic acid solution (Figure 3B,C). Chl *b* stabilizes LHCII assembly; its degradation initiates LHCII turnover.^41,42^ Three potential metabolic roles have been proposed for Chl *b* degradation in higher plants:^43–45^ (i) The degradation of Chl *b* helps remobilize nutrients. LHCII, a nutrient-rich chloroplast protein, should have its Chl *b* degraded first. Consequently, Chl *b* degradation affects nutrient remobilization between source tissues and various plant organs. (ii) A decrease in Chl *b* content reduces photodamage, as uncoupled LHCII can produce reactive oxygen species (ROS) if not properly degraded. Unregulated LHCII degradation during senescence is dangerous because it leads to excessive degradation and cell damage, disrupting the recovery of nutrients from leaves. (iii) Chl *b* degradation regulates the light-harvesting capacity of leaves. In chloroplasts, LHCII is the major antenna complex, and Chl *b* should be degraded before the overall level of LHCII decreases.

**Figure 3:**
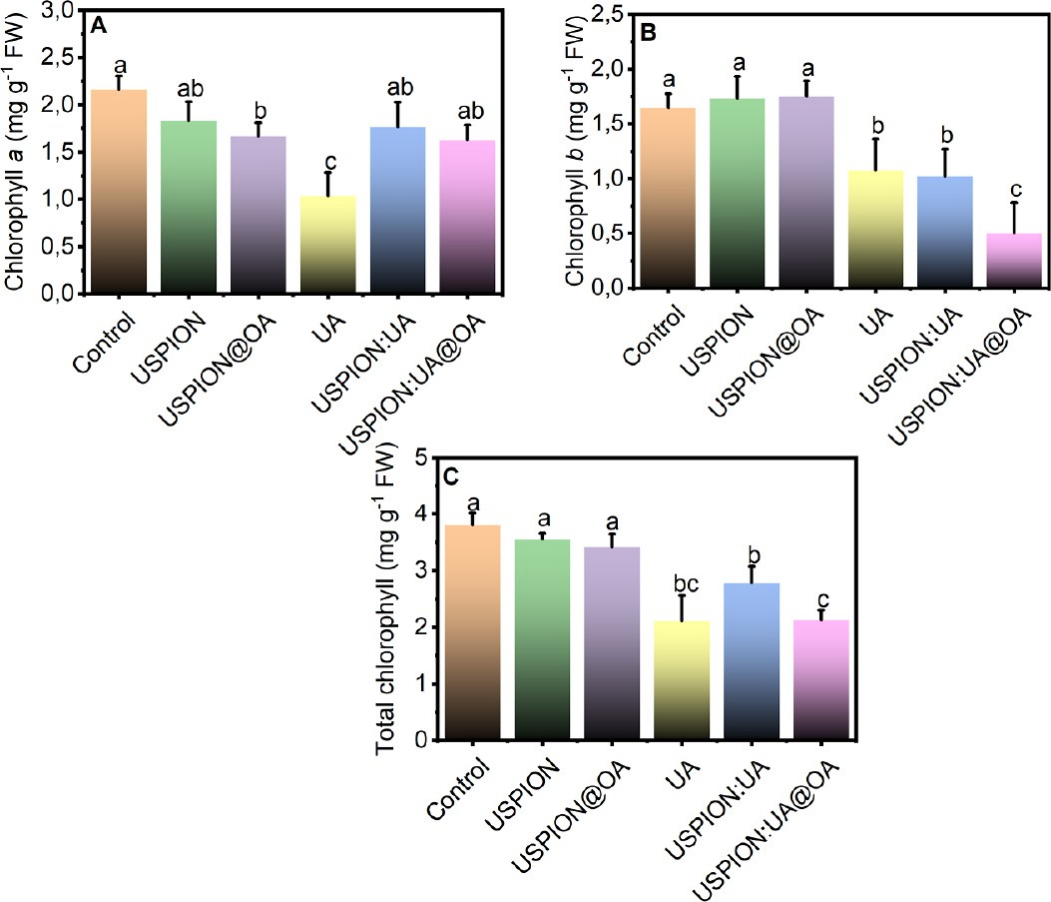
Concentrations of (A) chlorophyll a (Chl a), (B) chlorophyll b (Chl b), and (C) total chlorophyll in lettuce (*L. sativa* L.) leaves after exposure to 500 ppm of USPIONs, USPIONs@OA, UA, USPIONs/UA, and USPIONs@OA/UA. Data points show medians of *n* = 3, with error bars indicating standard error. Different letters denote significant differences among treatments by Kruskal–Wallis (*p* < 0.05).

#### 3.2.2 Photosystem II Photochemical Activity

Chlorophyll *a* fluorescence induction (Kautsky) curves were recorded to probe nanoparticle-induced alterations in the molecular organization and functional performance of the photosynthetic apparatus. The OJIP curves obtained from intact lettuce leaves are presented in Figure 4A, while the normalized fluorescence curves scaled to the Fo-to-Fm range are shown in Figure 4B as Vt = (Ft – Fo)/(Fm – Fo). The results showed that the presence of UA, USPIONs/UA, and USPIONs/UA@OA increases the J-step (∼2.0–3.0 ms), but the USPIONs and USPIONs@OA treatments did not change the J-step significantly, when compared to control samples. Our results suggest that UA bioherbicide and its nanoformulations are capable of completely inhibiting electron transfer in PSII (Figure 4B,C), attributed to Q_A_ (Q_A_^–^)^46^ accumulation stemming from perturbed redox transitions at P680^–^, Pheophytin^–^, or Q_A_^–^ levels.^30,35^ In this context, 1-V_J_ was significantly impaired in the lettuce leaves upon UA, USPIONs/UA, and USPIONs/UA@OA exposure, due to the blocked electron flow from Q_A_ → Q_B_ in the thylakoid PSII.^28^

**Figure 4:**
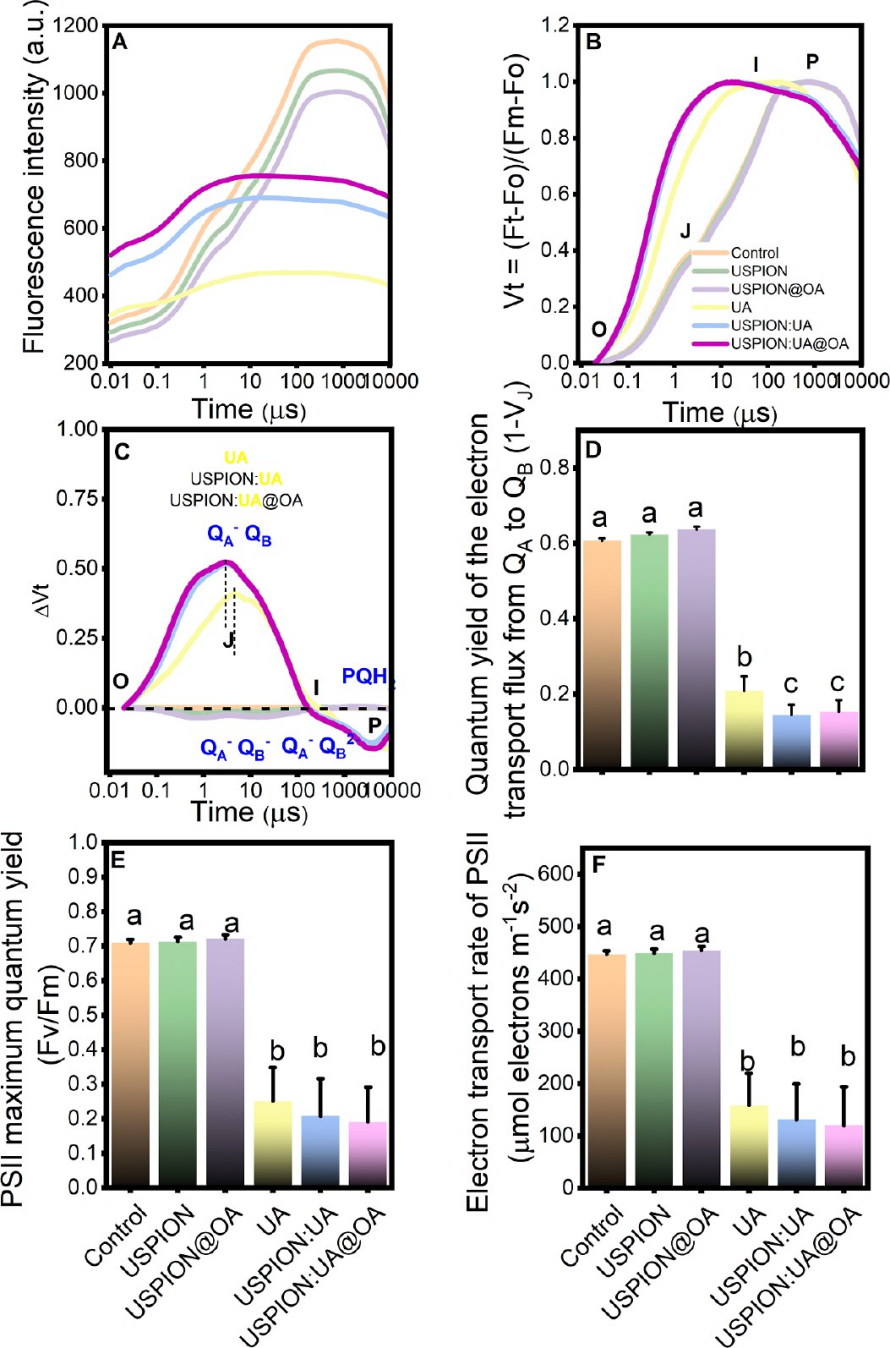
(A) *In vivo* chlorophyll *a* fluorescence induction and (B) relative variable chlorophyll *a* fluorescence (defined as *V*_OP_ = [(Ft – Fo)/(Fm – Fo)]) of the OJIP curves, (C) the difference of normalized fluorescence intensity as ΔVt, (D) quantum yield of electron transport flux from quinone A to quinone B (1 – *V*_J_), (E) maximum photochemical quantum yield of photosystem II (Fv/Fm), and (F) electron transport rate (ETR_PSII_). All parameters are presented for the plants exposed to 500 ppm of USPIONs, USPIONs@OA, UA, USPIONs/UA, and USPIONs/UA@OA treatments. Points denote medians of triplicates, with error bars denoting the standard error. Distinct letters indicate significant differences among treatments based on the Kruskal–Wallis test (*p* < 0.05). The measurements were conducted using a PEA hand-held fluorometer with red light (Ex λ 630 nm) excitation at 3.500 μmol m^–2^ s^–1^.

Our findings also revealed that combining our bioherbicide with magnetic nanocarriers significantly decreases the quantum yield of electron transport from Q_A_ to Q_B_. USPIONs/UA and USPIONs/UA@OA inhibited electron transport from Q_A_ to Q_B_ by approximately 30.35 and 26.41%, respectively, making them more effective than ionic usnic acid treatment (Figure 4D). Additionally, the ChlF data (*V*_t_ and Δ*V*_t_) indicated that the kinetics of electron transport flow inhibition from Q_A_ to Q_B_ varies over time (Figure 4C). UA exhibits a delayed time for reduction reaction events, increasing from 2.0 to 4.0 ms compared to the nanoenabled usnic acid formulations (USPIONs/UA and USPIONs/UA@OA). As anticipated, this delay in the time point of the photochemical reaction kinetics suggests that alterations in thylakoid photochemistry may result in different timings for the occurrence of these redox reactions.^47^ This statement suggests several important implications regarding the effects of the observed delay in photochemical reaction kinetics; our hypotheses are (i) the delay in photochemical reactions may indicate reduced efficiency in the photosynthetic process, potentially affecting the overall energy conversion; (ii) alterations in the timing of redox reactions can disrupt the flow of energy within the thylakoid membrane, potentially leading to an imbalance in energy transfer and usage; and (iii) delayed redox reactions can lead to the accumulation of intermediate products, increasing the risk of reactive oxygen species formation.

Gao and coauthors^18^ suggest that UA impairs photosynthetic activity in PSII primarily by reducing the electron transfer rate. Our data support this assertion, as evidenced by the significant accumulation of Q_A_^–^ when the electron fluxes beyond Q_A_ are disrupted.^48^ Additionally, UA nanoformulations further enhanced these target responses. Chlorophyll fluorescence analysis revealed that UA, USPIONs/UA, and USPIONs/UA@OA significantly reduced the Fv/Fm and ETR_PSII_ (Figure 4). Furthermore, the nanobioherbicide exhibited no significant differences from UA in these two parameters. Compared to the control and unloaded USPIONs, we suggest that UA and its nanoenabled formulations lead to a decrease in PSII photochemical activity by inhibiting the electron transport rate across the thylakoid membrane.

#### 3.2.3 Specific Energy Fluxes per Reaction Center in Thylakoid Membranes: A Network View

JIP-test parameters are shown in Figure 5, ABS/RC significantly increased with usnic acid and nanoenabled usnic acid treatments, while a weaker increase was observed with USPIONs capped with oleic acid (Figure 5A). These results suggest a decrease in the functioning of PSII reaction centers^18^ and indicate a potential side effect associated with the USPIONs@OA nanomaterial. On the other hand, DIo/RC did not differ between UA-free nanostructures and the control. However, DIo/RC was significantly increased upon usnic acid and the nanoformulations containing usnic acid treatments, while TRo/RC also increased (Figure 5B,C). The dissipated energy (DIo), which represents the energy lost by PSII reaction centers as fluorescence, heat, or through other pathways, along with the trapped energy fluxes per PSII reaction center that reflect the rate of excitons captured by open PSII RCs,^27^ suggests that our findings may indicate changes in the structural conformation of the antenna pigment–protein complexes. Our results indicate that lettuce leaves exposed to USPIONs/UA and USPIONs/UA@OA exhibited a greater reduction in the electron transfer rate from Q_A_ to Q_B_ in the thylakoid membranes compared to those treated with UA (Figure 5D). Note that nanoparticles without UA do not affect the electron transport flux in the chloroplast. Given that the nanoenabled usnic acid formulations can inhibit the PSII electron transport at the quinone level, the decreased energy fluxes from PSII to the acceptor side of PSI (REo/RC) result from the inhibition of the electron transfer rate from Q_A_ to Q_B_ (Figure 5E). It is found that the fraction of Q_A_-reducing center reduced highly when leaves are treated with UA, USPIONs/UA, and USPIONs/UA@OA, respectively (Figure 5F).

**Figure 5:**
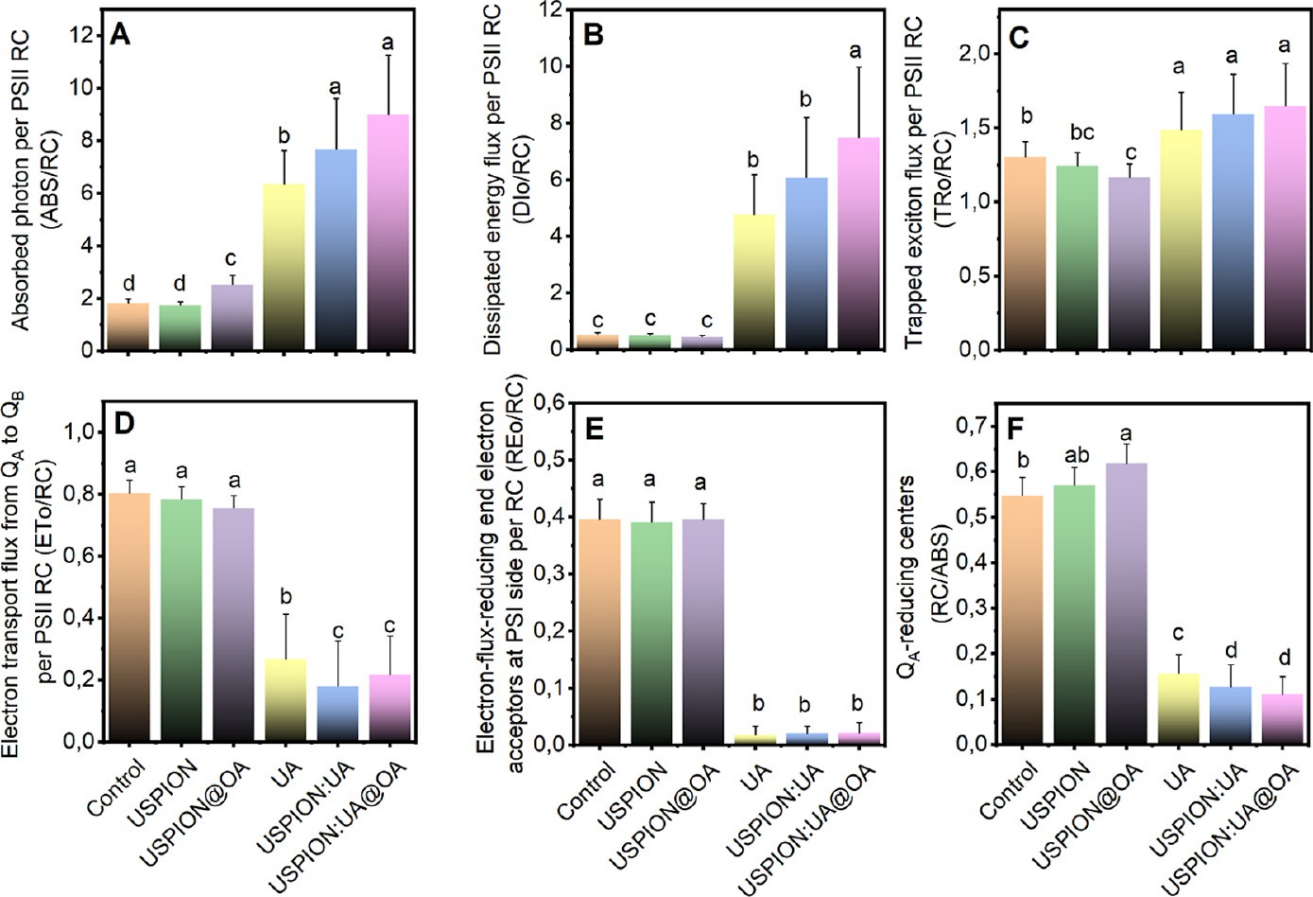
Specific energy fluxes (per reaction center, RC) for (A) energy absorption (ABS/RC), (B) dissipated energy (DIo/RC), (C) trapped energy (TRo/RC), (D) electron transport flux (ETo/RC), (E) electron flux-reducing end electron acceptors at PSII side (REo/RC), and (F) Q_A_-reducing centers (RC/ABS). All parameters are presented for the plants exposed to 500 ppm of USPIONs, USPIONs@OA, UA, USPIONs/UA, and USPIONs/UA@OA treatments. Data points represent the median of triplicate measurements, with error bars denoting the standard error. Distinct letters indicate significant differences among treatments based on the Kruskal–Wallis test (*p* < 0.05). The measurements were conducted using a PEA hand-held fluorometer with red light (Ex λ 630 nm) excitation at 3.500 μmol m^–2^ s^–1^.

The data indicated that USPIONs/UA and USPIONs/UA@OA are indeed most effective to impair the RCs of PSII than UA. Additionally, nanobioherbicide-induced changes in the energy fluxes parameters can also be assessed by the thylakoid membrane pipeline model.^27^ Parameter changes are depicted by the differing widths of the corresponding arrows (Figure 6). In summary, the absorption flux (ABS), trapping flux (TRo), and dissipated energy flux (DIo) clearly increased with UA, USPIONs/UA, and USPIONs/UA@OA treatments. The electron transport flux (ETo), and amount of active PSII RC declined significantly. UA, and consequently its nanoformulations, act by disabling PSII RC through inhibition of electron flow at quinone acceptor sites, coupled with disruption of pigment complexes in the light-harvesting antenna.

**Figure 6:**
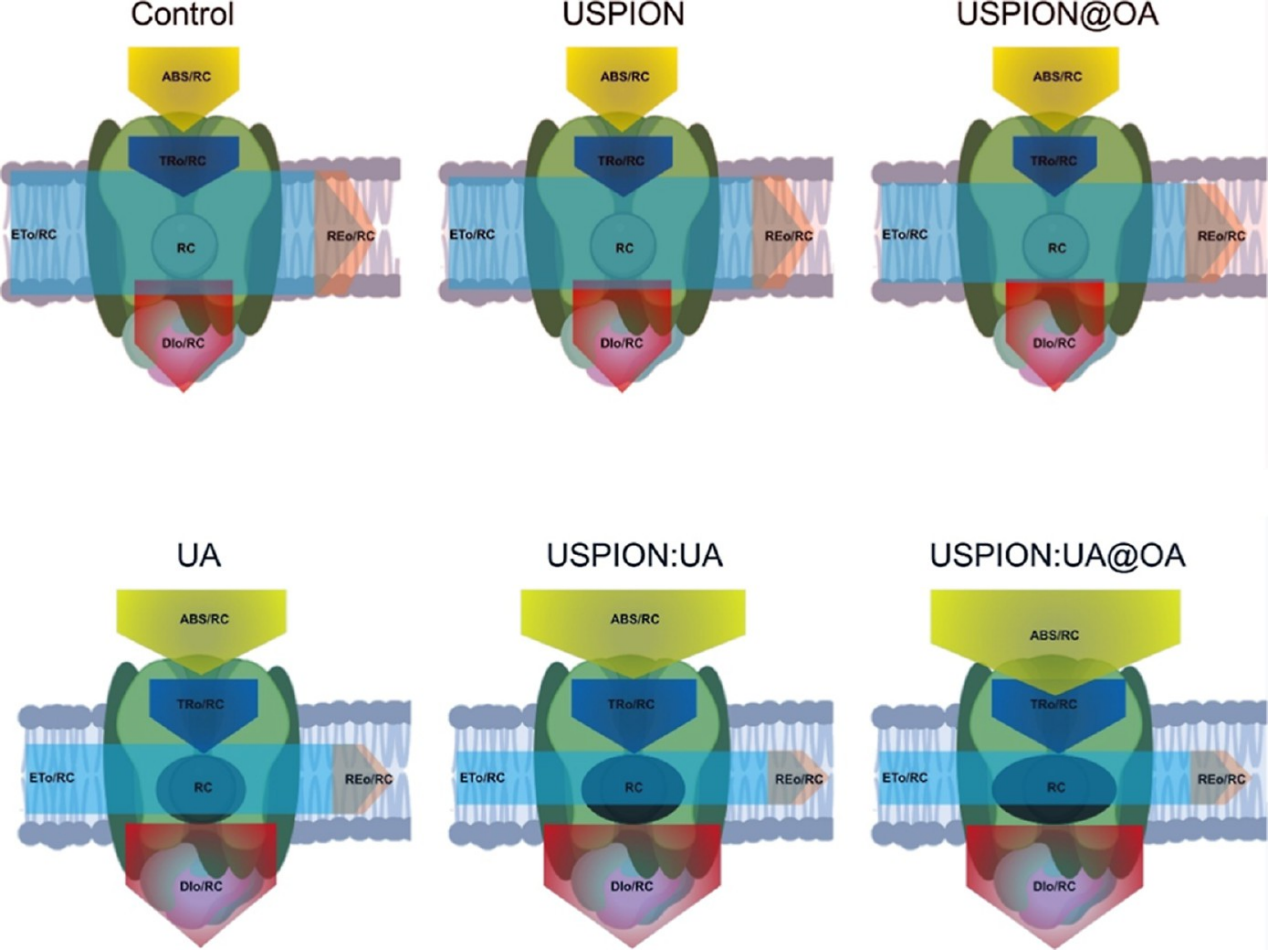
Energy pipeline membrane models of specific energy fluxes (per reaction center, RC). Parameter magnitudes are conveyed by the corresponding arrow widths; open versus solid black circles indicate the proportions of active (Q_A_-reducing) and inactive (non-Q_A_-reducing) PSII reaction centers, respectively. ABS/RC, photon flux absorbed by the antenna pigments per RC; TR/RC, trapped energy flux per RC; ET/RC, electron transport flux per RC; DIo/RC, nonphotochemical energy dissipation; and REo/RC: electron flux leading to a reduction in the PSI end acceptor (RE_0_/RC). The measurements were conducted using a PEA hand-held fluorometer with red light (Ex λ 630 nm) excitation at 3.500 μmol m^–2^ s^–1^.

The maintenance of photosynthetic-specific energy flux per reaction center in thylakoid membrane systems depends on adaptive responses that reflect the system’s homeostatic ability to manage exogenous disturbances.^49–51^ Conceptual models are often required to understand such complex phenomena in nature.^52^ Figure 7A displays Pearson correlation coefficients (r) for each paired variable within the network of specific energy fluxes in PSII thylakoid membranes.^13,30^ The Pearson’s correlogram plots (Figure 7A) reveal differences in the strength of relationships across variable pairs, indicating that treatments with USPIONs, USPIONs@OA, UA, USPIONs/UA, and USPIONs/UA@ can modulate the photochemical network of specific energy fluxes at PSII thylakoid membrane systems.

**Figure 7:**
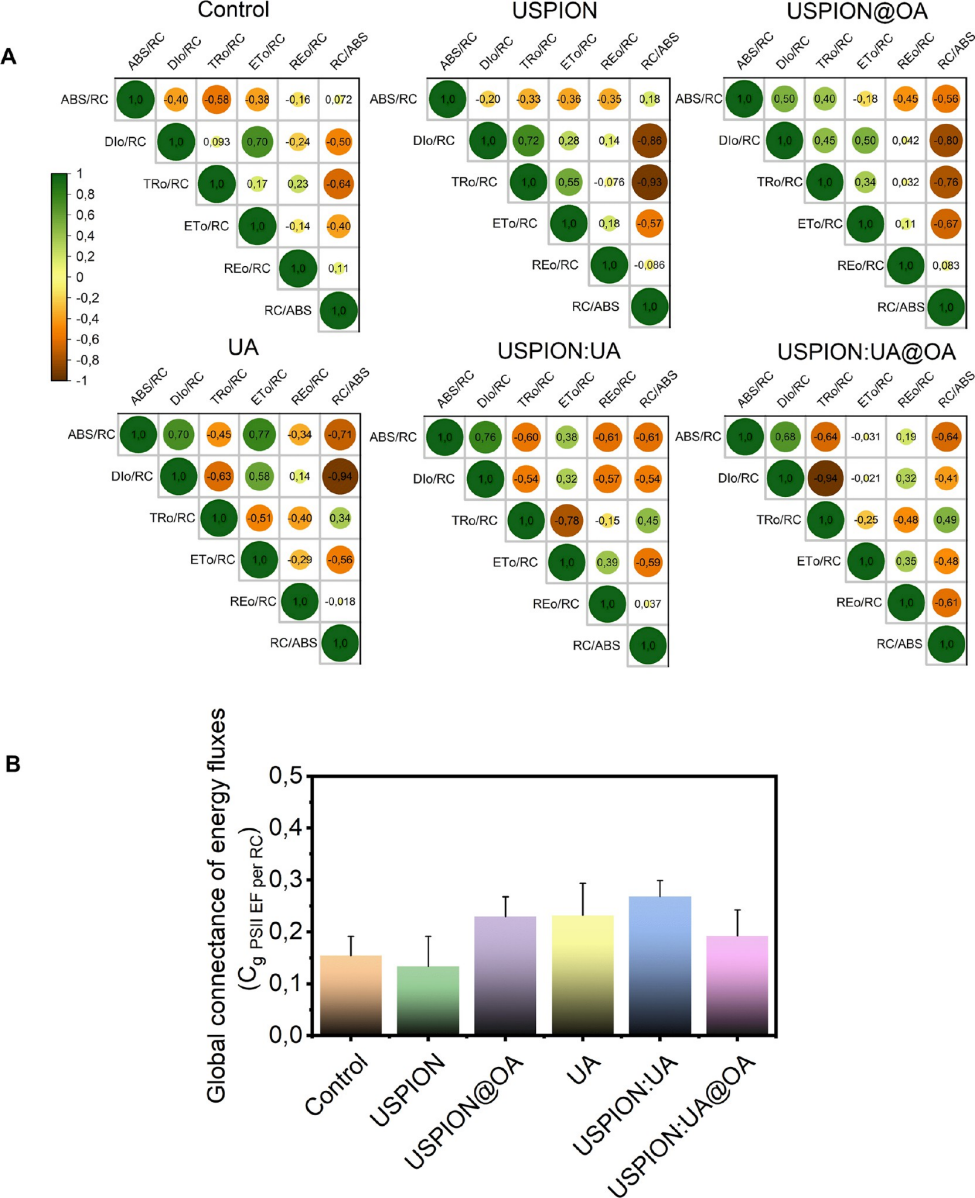
(A) Pearson correlogram of energy fluxes (per reaction center, RC) of photosystem II, reflecting the connectance among their paired parts in a network and (B) Global connectance index for PSII energy fluxes, quantifying overall network integration. The measurements were conducted using a PEA hand-held fluorometer with red light (Ex λ 630 nm) excitation at 3.500 μmol m^–2^ s^–1^.

A significant alteration was observed in the global connectivity of the photochemical energy flux network (Figure 7B). Network connectance analysis, a method rooted in systems biology, provides insight into how complex interactions evolve and offers a holistic view of biological responses to external stimuli.^51^ Our results indicate that ultrasmall magnetic nanoparticles carrying UA disrupt photochemical energy fluxes and the connectance among the network elements. To better understand how nanoherbicides targeting PSII inhibition interact with photochemical systems, further research is needed to investigate nonlinear interactions within the photosynthetic light reaction network as a complex system.

High network connectance may reduce the quality of specific energy fluxes, as shown in Figure 7B for USPIONs@OA, UA, USPIONs/UA, and USPIONs/UA@OA, which may indicate low modularity within the PSII energy flux network. External stimuli trigger processes that forge interaction links within the network.^53^ Emergent properties of whole photochemical networks can be analyzed using system-level metrics, such as connectance^51,54^ to further elucidate the functioning and stability of biological systems.^55^ Photostasis is essential for photoautotrophic organisms, enabling them to balance light-derived energy to sustain cellular homeostasis. This process regulates light absorption, energy transfer, and dissipation to optimize photosynthesis, especially under stress conditions, such as excessive or insufficient light and other environmental stressors.^56^ Under stress, photoautotrophs adjust their photostasis mechanisms to protect themselves, ensuring survival and cellular function.

#### 3.2.4 Transcriptional Regulation of Photosystem Marker Genes

Real-time qPCR was employed to quantify the transcript levels of four target genes in this study; the PSII D1 protein, encoded by psbA, forms an essential structural component of the PSII complex. Notably, USPIONs and oleic acid-capped USPIONs showed approximately 4-fold and 11-fold increases in psbA transcript levels, respectively, compared to control treatment (Figure 8A). Furthermore, the UA and UA nanoformulations led to significant increases in *psbA* gene expression relative to that of the control (Figure 8A). The observed overexpression suggests that an increased abundance of D1 protein in the thylakoid membrane could enhance PSII’s photon capture capability during electron transport, even when this transport is partially inhibited by the nanobioherbicide. The *psbA* gene has also been associated with herbicide tolerance, where specific point mutations correlate with varying tolerance levels.^57–59^ These transcriptional findings suggest that lettuce may overexpress *psbA* as a defense response to usnic acid and nanoenabled formulations.

**Figure 8:**
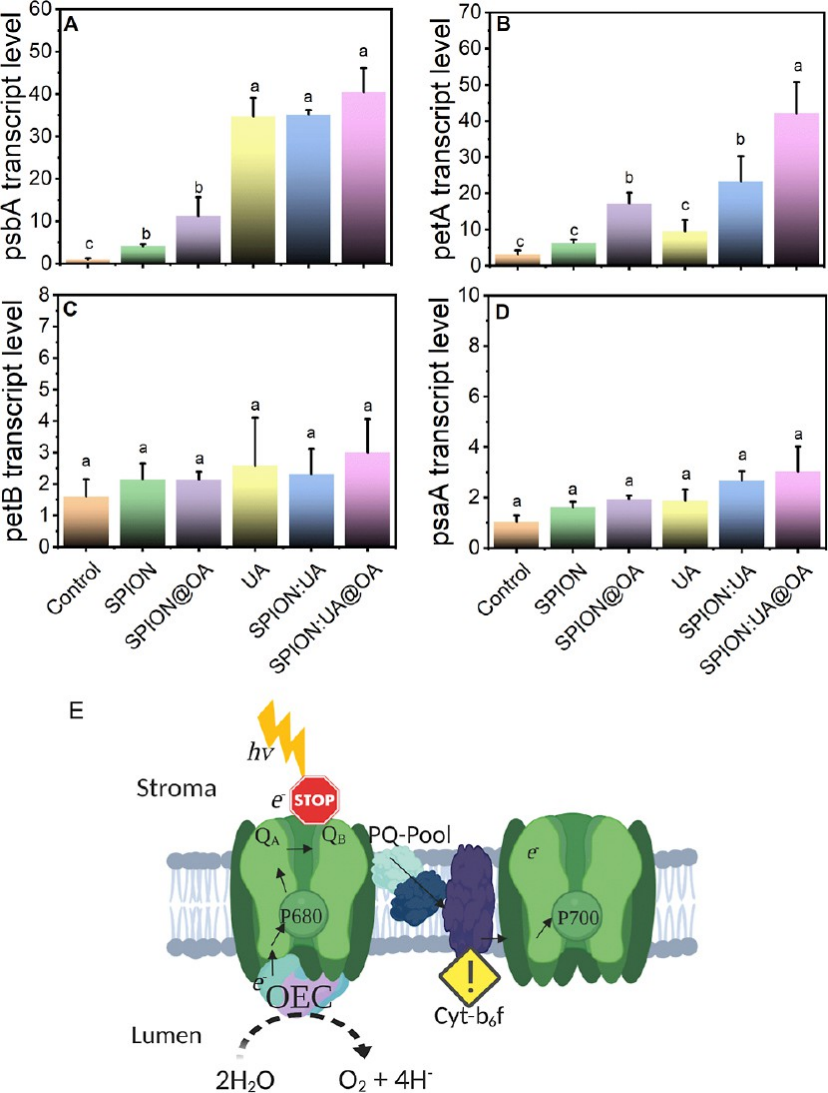
Lettuce (*L. sativa* L.) photosystem gene expression: relative transcript abundances. All evaluated parameters are displayed for the 500 ppm treatments of USPIONs, USPIONs@OA, UA, USPIONs/UA, and USPIONs/UA@OA. (A) Photosystem II reaction center protein A, psbA, (B) photosynthetic electron transfer A, petA, (C) photosynthetic electron transfer B—cytochrome b6, petB, and (D) photosystem I P700 chlorophyll A apoprotein A1, psaA. Gene expression was quantified by real-time RT-qPCR as the mean of three independent replicates, with normalization to the 18S rRNA reference gene. (E) Schematic representation of the potential photoinduced inhibitory mechanisms of our nanobioherbicide on the photosynthetic apparatus at the thylakoid membrane level, illustration created with BioRender.com. Created by the authors.

Nanomaterials are known to alter the mechanisms of action and structural properties of pesticides.^60^ The cytochrome b6f (cytb6f) complex, encoded by the petA gene, is crucial in photosynthesis, coupling electron transfer between PSI and PSII with proton motive force (PMF) generation via the Q-cycle.^61,62^ The USPIONs/UA and USPIONs/UA@OA formulations upregulate *petA* expression (Figure 8B), likely due to the disruption of Q_A_ → Q_B_ electron transport. The observed upregulation may contribute to a metabolic response that maximizes free radical and ROS production in the chloroplast.^63^ On the other hand, usnic acid solution alone represses *petA* expression. The nanoparticle delivery system does not significantly impact the expression of PSII genes *petB* and *psaA* (Figure 8C,D), which showed no transcriptional changes. The proposed thylakoid inhibition mechanism of our nanobioherbicide is illustrated in Figure 8E.

### 3.3 Computational Insight into Probe Action Target of Magnetic Bioherbicide Nanoparticle

As shown in Figure 9, USPIONs and usnic acid were docked with the D1 protein. USPION was predicted to interact near the center of the D1 protein, while UA showed interactions close to that of the Q_B_ catalytic domain. The molecular docking mechanisms of UA near the Q_B_ binding niche align with our experimental results (Figure 4A–D). The inhibition of electron flow in thylakoid PSII from Q_A_ to Q_B_ observed in lettuce leaves may be related to UA’s interaction with amino acid residues adjacent to the Q_B_ catalytic domain. These findings collectively suggest that UA has a strong potential as a new herbicide targeted to photosystem II inhibition. Similar interactions have been observed for various herbicides and bioherbicides that target PSII inhibition, including atrazine, diuron, metribuzin, terbuthylazine, metobromuron, bentazon, and natural albequines.^17,59,64^

**Figure 9:**
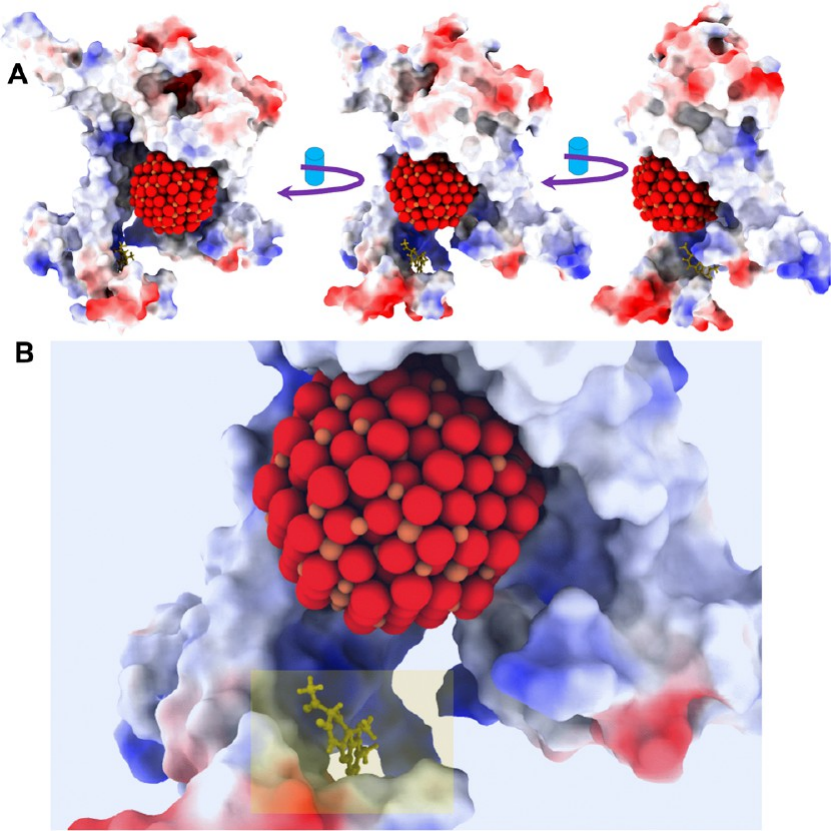
Molecular docking between the ultrasmall superparamagnetic iron oxide nanoparticles (USPIONs) and usnic acid (UA) with the lettuce D1 protein of photosystem II. (A) Lettuce D1 protein of photosystem II depicting the USPION and UA deposition to their catalytic and Q_B_ binding niche domains, respectively. (B) Inset details of USPION and UA deposition D1 protein.

The UA molecule was docked next to the Q_B_ binding niche. The docking analysis showed that UA presents hydrogen bonds with His 205, Glu 234, Thr 235, and His 262 amino acid residues. Additionally, negatively charged and polar interactions are observed in this study around the Q_B_ active site. In this regard, it is possible to suggest a disturbance promoted by UA in this protein (Figure S5 Supporting Information), compromising Q_A_ → Q_B_ electron transfer through competition with the quinone substrate for the catalytic site,^65^ via competitive interaction with the quinone substrate to the catalytic pocket.^66^ The highest binding affinity was observed at the D1 protein’s Q_B_ pocket, where UA established four hydrogen-bonds involving His205, Glu234, Thr235, and His262 residues. Histidine is related to be an important residue related to herbicide resistance in PSII.^59^ Our findings imply a key role in histidine residues on the UA binding behavior, probably due to the protonation state of histidine amino acid.^67^

### 3.4 Stress-Related Antioxidant Enzyme Activity

Oxidative stress responses in lettuce were assessed by the antioxidant machinery comprised of SOD, CAT, and GSH. Our findings, as illustrated in Figure 10A, revealed a noteworthy upregulation (*p* < 0.05) in SOD activity in plants treated with UA, USPIONs/UA, and USPIONs/UA@OA when compared to leaves treated with UA-free particles. Notably, SOD activity was significantly diminished in plants treated with USPIONs@OA. SOD is a key antioxidant enzyme that mitigates the damaging effects of reactive oxygen species in biological systems. Through its enzymatic activity, SOD efficiently converts the superoxide radical (O_2_^●–^) into hydrogen peroxide (H_2_O_2_), which serves as a less toxic ROS.^22,35,60^

**Figure 10:**
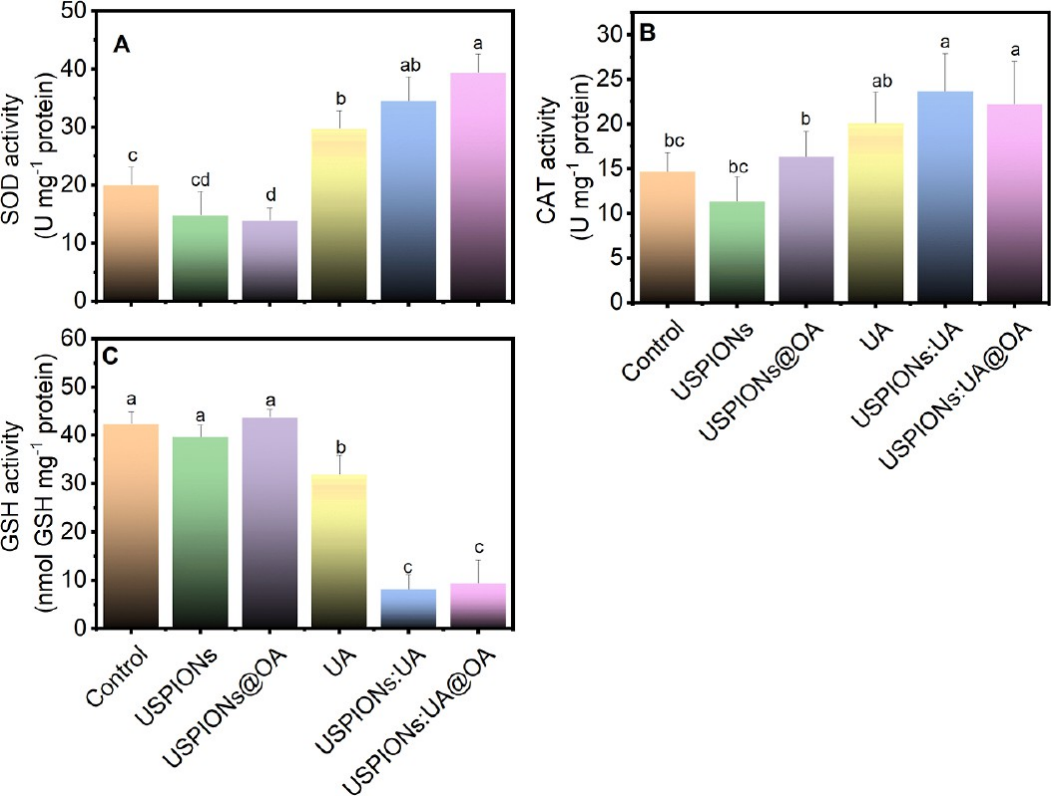
(A) Superoxide dismutase (SOD), (B) catalase (CAT), and (C) glutathione (GSH) activity in lettuce leaves exposed to 500 ppm of USPIONs, USPIONs@OA, UA, USPIONs/UA, and USPIONs/UA@OA treatments. Different letters mark groups differing from the control by Dunnett’s test (*p* < 0.05). Data are reported as mean ± SD (*n* = 5).

The H_2_O_2_ can then be enzymatically decomposed by other antioxidant enzymes such as CAT and GSH into water (H_2_O), completing the detoxification process.^68^ Similarly, CAT activity displayed a significant rise (*p* < 0.05) in response to treatment with USPIONs/UA and USPIONs/UA@OA when compared to the control (Figure 10B). Conversely, the activity of GSH decreased in lettuce leaves treated with USPIONs/UA and USPIONs/UA@OA. These findings suggest that the application of our nanobioherbicide may influence the activity of key antioxidant enzymes, critical components of the cellular antioxidant defense system (Figure 10C).

These cascades of enzymatic reactions orchestrated by SOD, CAT, and GSH serve as a critical protective mechanism to oxidative stress, safeguarding cells from ROS-induced damage.^60^ The intricate interplay among these antioxidant enzymes underscores the sophisticated, multifaceted nature of the cellular antioxidant defense system and its central role in sustaining redox homeostasis while shielding against oxidative injury.^69,70^ Further studies are needed to clarify the mechanisms behind these findings and their implications, guiding the design of safer, more effective nanobioherbicides for sustainable agriculture.

## 4 Conclusion

Collectively, the data reveal that the target effects of an ultrasmall magnetic nanoparticle loaded with a bioherbicide on PSII activity demonstrate significant advances in ultrasmall magnetic nanoparticle systems carrying UA as chemical cargo. The usnic acid-loaded USPION formulation showed favorable physicochemical profiles, stabilized smaller particle sizes with well-defined morphology, and exhibited sustained, stimulus-responsive release behavior. These excellent properties make it suitable for potential agricultural use. Furthermore, the USPIONs/UA and USPIONs/UA@OA enhanced the inhibition of photosynthetic electron transport chain at the quinone level in PSII, demonstrating that the ultrasmall magnetic nanoplatform preserved its mechanism of action and achieved higher efficacy than its unloaded counterpart.^13^ Gene expression analysis revealed an increased level of induction of the photosynthetic genes *psbA* and *petA*. Molecular docking analysis showed USPIONs and UA anchored to D1 with stable orientations beside the Q_B_ binding niche, consistent with UA intercepting electrons during the electron flow from Q_A_ to Q_B_. The alterations in enzyme activity observed in this study highlight the upregulation of SOD in lettuce leaves upon treatment with UA, USPIONs/UA, and USPIONs/UA@OA. Moreover, CAT activity increased to a comparable extent across those conditions in leaves treated with both nanoformulations incorporating usnic acid. Interestingly, a noteworthy decrease in the GSH activity was also observed for these treatments. These findings indicate that a nanobioherbicide can modulate key antioxidant enzymes that are central to the cellular antioxidant defense system. Our results indicate that the nanoformulation increased PSII-specific inhibition relative to free UA, warranting field-level validation and ecotoxicological assessment before translational claims in precision agriculture can be substantiated.

## Supplementary Material



## Notes

Dose-dependent maximum photochemical quantum yield of photosystem II (Fv/Fm) values; protein structure homology model of target protein generated using SwissModel server; ligand structures used in molecular docking studies including usnic acid and Fe_3_O_4_ nanoparticle with 408 atoms; X-ray diffraction patterns of crystalline magnetite nanoparticles confirming inverse spinel structure; average crystallite size and dislocation density data for all nanocomposite materials; and molecular docking results showing USPION and usnic acid binding sites on D1 protein subunit of photosystem II (PDF)
