# Supplementary material for: Targeted Inhibition of Photosystem II Electron Transport Using Bioherbicide-Loaded Ultrasmall Nanodevices
Source: ACS Omega. 2025 Nov 17;10(46):55733–49. doi: 10.1021/acsomega.5c07085 (PMC12658649; doi:10.1021/acsomega.5c07085)
Supplement: Supplementary file 1 [file ao5c07085_si_001.pdf]

Supplementary data

Targeted inhibition of photosystem II electron transport using bioherbicide-loaded ultrasmall nanodevices

Montcharles S. Pontes [a,b*], Leandro O. Araujo [b], Jaqueline S. Santos [c], José Luiz da Silva [d], Thaiz B.A.R. Miguel [e], Emilio C. Miguel [e], Sandro M. Lima [a], Luís H.C. Andrade [a], Gilberto J. Arruda [a], Jean-Claude M'Peko [f], Samuel L. Oliveira [b], Renato Grillo [g], Anderson R.L. Caires [b], Etenaldo F. Santiago [a*]

[a] *Plant Resources Study Group, Center for Natural Resources Study (CERNA), Mato Grosso do Sul State University (UEMS), Dourados, MS, Brazil*
[b] *Optics and Photonics Group, SISFOTON Lab, Institute of Physics, Federal University of Mato Grosso do Sul (UFMS), Campo Grande, MS, Brazil*
[c] *Genetics Department, Luiz de Queiroz College of Agriculture (ESALQ), University of São Paulo (USP), Piracicaba, SP, Brazil*
[d] *Department of Analytical, Physico-Chemical and Inorganic Chemistry, Institute of Chemistry, São Paulo State University (UNESP), Araraquara, 14800-060, Brazil*
[e] *Laboratory of Biomaterials, Department of Metallurgical and Materials Engineering, Federal University of Ceará (UFC), Fortaleza, CE, Brazil*
[f] *São Carlos Institute of Physics, University of São Paulo (USP), São Carlos, SP, Brazil.*
[g] *Environmental Nanochemistry Group, Department of Physics and Chemistry, São Paulo State University (UNESP), Ilha Solteira, SP, Brazil*

Corresponding author:

*E-mail: felipe@uems.br (E.F. Santiago)

*E-mail: montcharles.pontes@gmail.com (M.S. Pontes)

Photosystem II inhibition

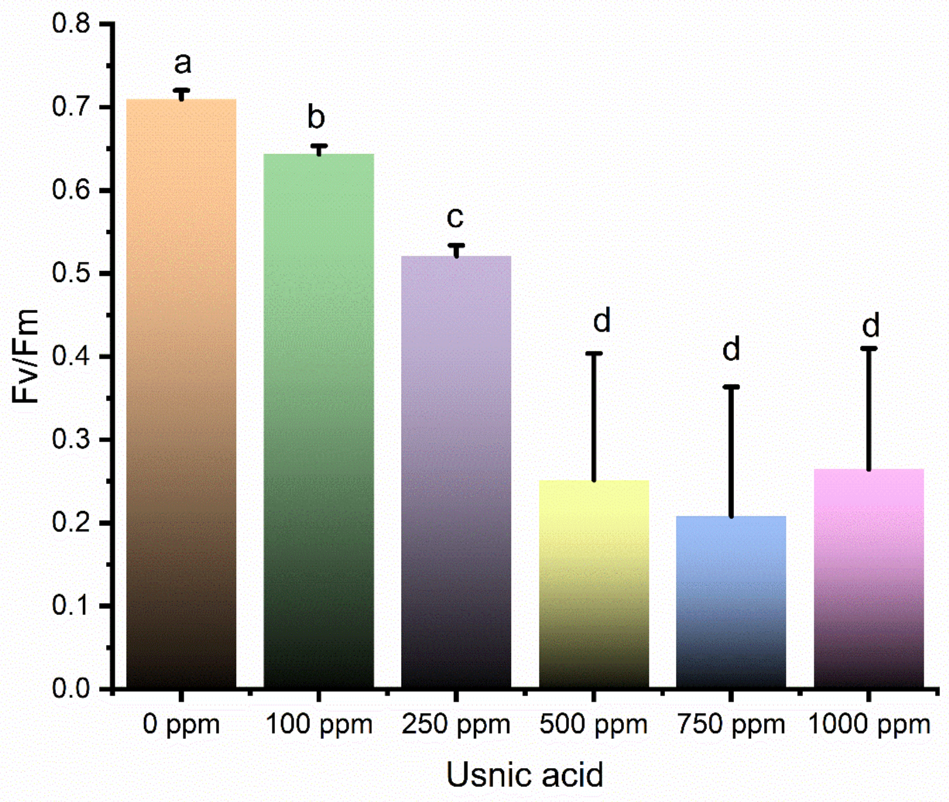

Figure S1. Dose dependent maximum photochemical quantum yield of photosystem II (Fv/Fm) values.

Protein modeling

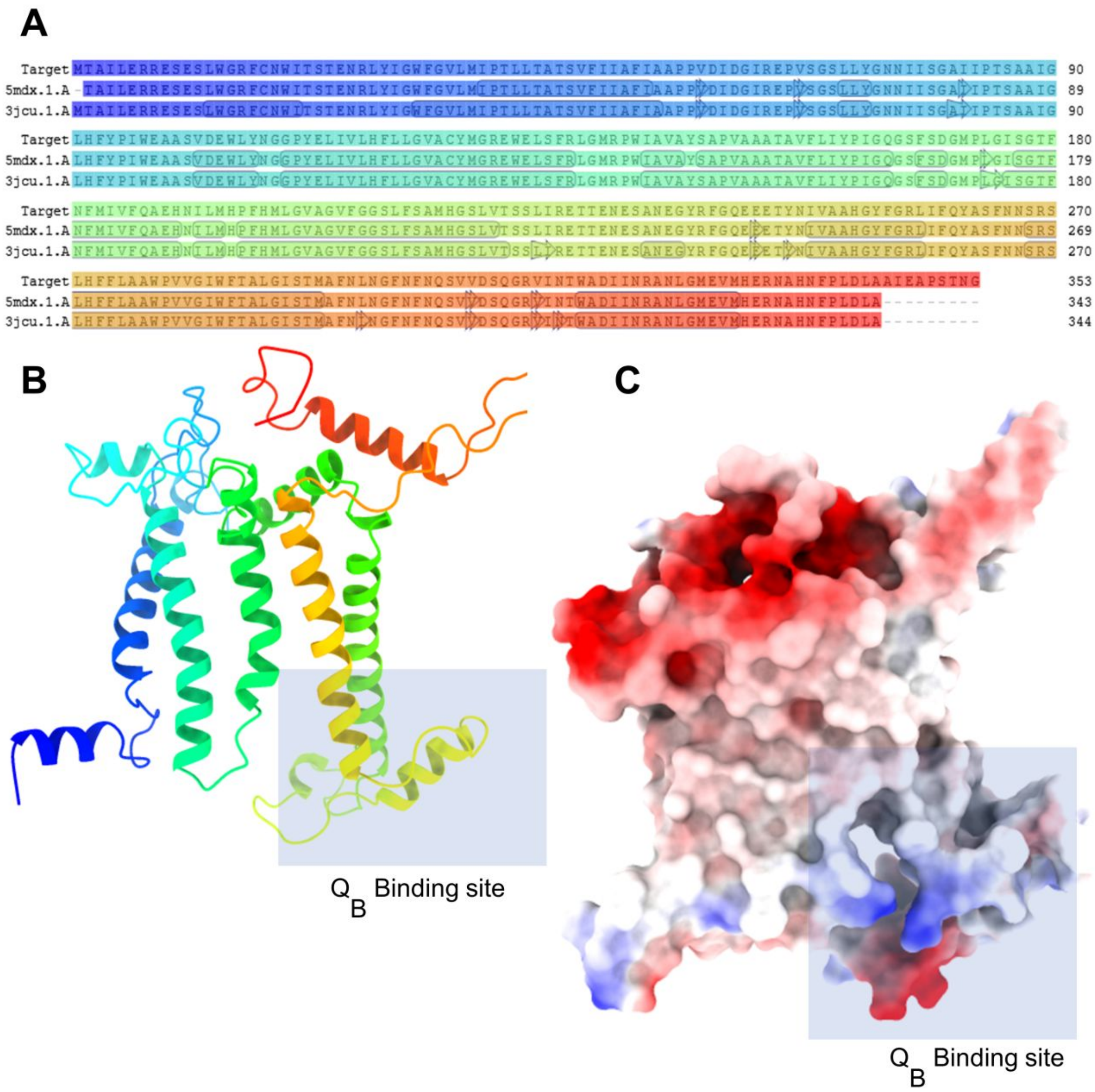

Figure S2. (A) Protein sequence alignment, (B) homology model structure, and (C) Q_B binding site visualization of the target protein modeled using the protein structure homology-modeling server SwissModel.

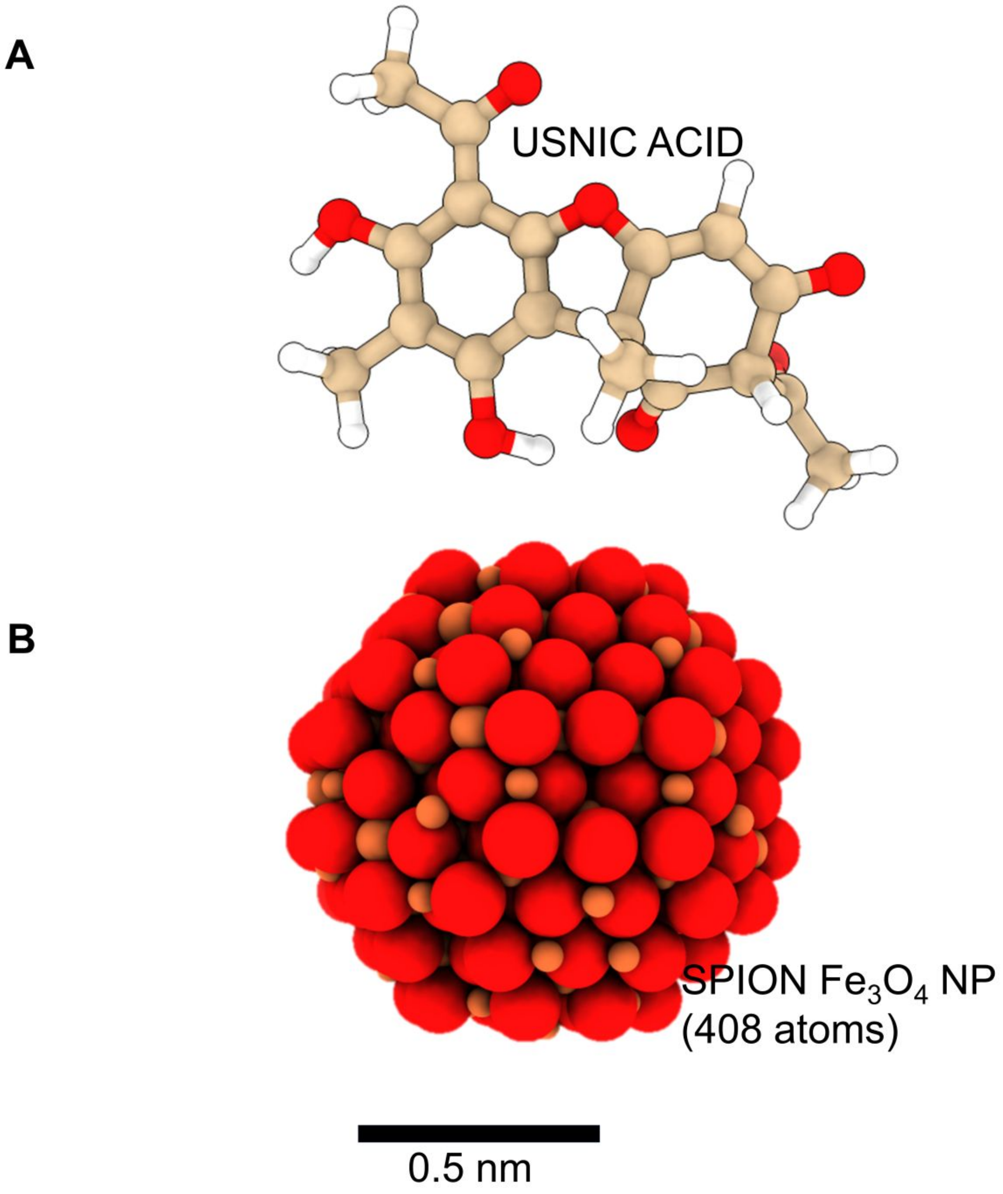

Figure S3. Ligands used in this study. (A) Usnic acid, and (B) Fe_3O_4 nanoparticle with 408 atoms and 1 nm diameter size.

X-ray diffraction patterns

Table S1 summarizes the average crystallite size (DD) and dislocation density of the crystalline magnetite nanoparticles as determined by X-ray diffraction (XRD) analysis. The presented data demonstrate that all the studied nanocomposite materials exhibit crystallite sizes in the nanometer range, varying between approximately 6 and 8 nm, with respective dislocation densities that corroborate their highly crystalline nature. These quantitative outcomes provide robust evidence of successful synthesis and structural control across the different nanoparticle formulations. Figure S4 displays the XRD patterns of the magnetite nanoparticles, revealing sharp and well-defined diffraction peaks characteristic of the inverse spinel structure typical of magnetite (Fe_3O_4). Collectively, these results confirm the preservation of the crystalline magnetite phase throughout all sample preparations.

Table S1. Average crystallite size (D), and dislocation density (δ) of crystalline magnetite nanoparticles (SPION, SPION:UA, SPION@OA, and SPION@OA:UA). Crystalline nature was attributed based on the XRD analysis.

Nanocomposite	*D* (nm)	δ (nm^{-2})
USPION	5.96	2.81×10^{-4}
USPION:UA	8.33	1.44×10^{-4}
USPION@OA	7.73	1.84×10^{-4}
USPION@OA:UA	7.51	1.77×10^{-4}

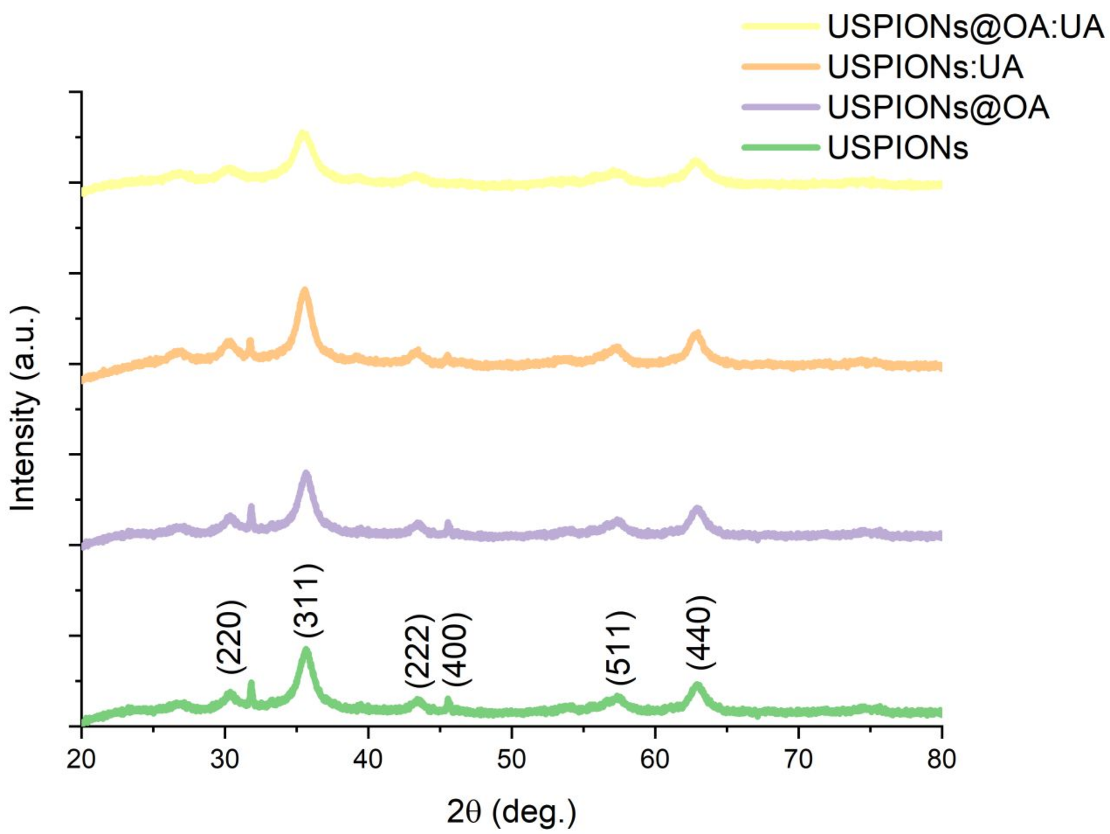

Figure S4. X-ray diffraction patterns of magnetite nanoparticles with crystalline structure (USPIONs, USPION@OA, SPION:UA, and SPION@OA:UA, respectively).

Molecular docking

For USPION-PSII D1 protein, the binding region is surrounded by Leu 41, Ala 44, Thr 45, Phe 48, Ile 49, Phe 52, Ile 77, Ile 78, Ile 115, Phe 119, Val 123, Tyr 126, Arg 129, Glu 130, Tyr 147, Pro 150, Val 151, Ala 153, Ala 154, Val 157, Phe 158, Met 172, Pro 173, Leu 174, Gly 175, Ile 176, Thr 179, Phe 180, Val 205, Ser 212, and Ile 283 as neighbouring residue (Fig. S5). Also, the preferable binding of USPION to D1 protein in PSII complex obtained was through non-polar and aromatic residues bond formation.

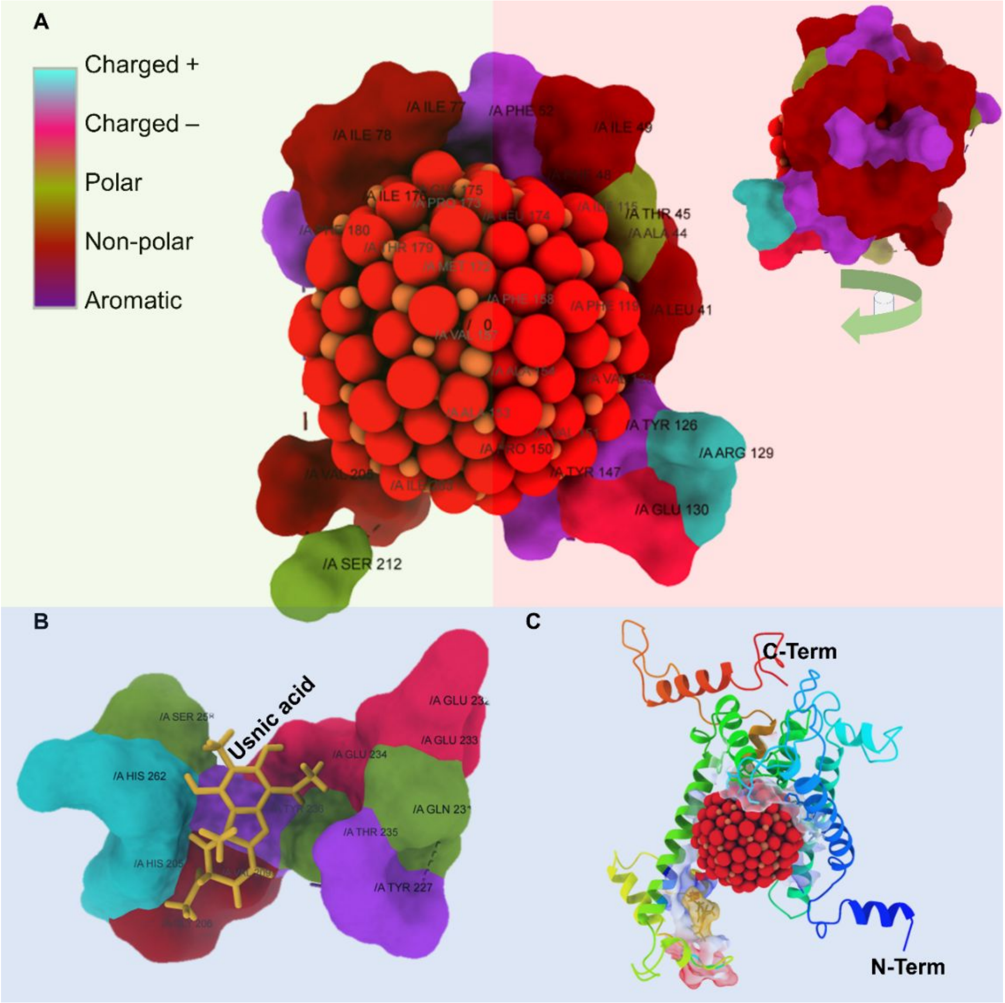

Figure S5. USPION and usnic acid docked onto amino acid residues of D1 protein. A) USPION surrounded by D1 protein amino acid residues, B) usnic acid docked next to Q_B binding niche, and C) USPION and usnic acid docked onto D1 subunit protein of photosystem II from lettuce.
